# Integrated Analysis of circRNA-miRNA-mRNA Regulatory Networks in the Intestine of *Sebastes schlegelii* Following *Edwardsiella tarda* Challenge

**DOI:** 10.3389/fimmu.2020.618687

**Published:** 2021-01-20

**Authors:** Min Cao, Xu Yan, Baofeng Su, Ning Yang, Qiang Fu, Ting Xue, Lin Song, Qi Li, Chao Li

**Affiliations:** ^1^ School of Marine Science and Engineering, Qingdao Agricultural University, Qingdao, China; ^2^ College of Marine Science and Biological Engineering, Qingdao University of Science & Technology, Qingdao, China; ^3^ School of Fisheries, Aquaculture and Aquatic Sciences, Auburn University, Auburn, AL, United States

**Keywords:** *Sebastes schlegelii*, *Edwardsiella tarda*, intestine, circRNA-miRNA-mRNA network, immune response

## Abstract

*Sebastes schlegelii*, an important aquaculture species, has been widely cultured in East Asian countries. With the increase in the cultivation scale, various diseases have become major threats to the industry. Evidence has shown that non-coding RNAs (ncRNAs) have remarkable functions in the interactions between pathogens and their hosts. However, little is known about the mechanisms of circular RNAs (circRNAs) and coding RNAs in the process of preventing pathogen infection in the intestine in teleosts. In this study, we aimed to uncover the global landscape of mRNAs, circRNAs, and microRNAs (miRNAs) in response to *Edwardsiella tarda* infection at different time points (0, 2, 6, 12, and 24 h) and to construct regulatory networks for exploring the immune regulatory mechanism in the intestine of *S. schlegelii.* In total, 1,794 mRNAs, 87 circRNAs, and 79 miRNAs were differentially expressed. The differentially expressed RNAs were quantitatively validated using qRT-PCR. Kyoto Encyclopedia of Genes and Genomes (KEGG) enrichment analysis showed that most of the differentially expressed mRNA genes and the target genes of ncRNAs were related to immune signaling pathways, such as the NF-κB signal pathway, pathogen recognition receptors related to signaling pathways (Toll-like receptors and Nod-like receptors), and the chemokine signaling pathway. Based on these differentially expressed genes, 624 circRNA-miRNA pairs and 2,694 miRNA-mRNA pairs were predicted using the miRanda software. Integrated analyses generated 25 circRNA-miRNA-mRNA interaction networks. In a novel_circ_0004195/novel-530/*IκB* interaction network, novel_530 was upregulated, while its two targets, novel_circ_0004195 and *IκB*, were downregulated after *E. tarda* infection. In addition, two circRNA-miRNA-mRNA networks related to apoptosis (novel_circ_0003210/novel_152/apoptosis-stimulating of p53 protein 1) and interleukin (novel_circ_0001907/novel_127/interleukin-1 receptor type 2) were also identified in our study. We thus speculated that the downstream NF-κB signaling pathway, p53 signaling pathway, and apoptosis pathway might play vital roles in the immune response in the intestine of *S. schlegelii.* This study revealed a landscape of RNAs in the intestine of *S. schlegelii* during *E. tarda* infection and provided clues for further study on the immune mechanisms and signaling networks based on the *circRNA-miRNA-mRNA axis* in *S. schlegelii*.

## Introduction

The immune system of vertebrates is a complex network that consists of different types of molecules, cells, and organs and plays key roles in recognizing foreign “invaders,” such as bacteria and viruses, thus resisting pathogen invasion and maintaining homeostasis (1). Distinct from other invertebrates, fish live in aquatic environments enriched with microorganisms. The mucosal surfaces, including the intestine, skin, and gills, serve as the first line of host defense, forming an immune barrier once the organism is invaded by pathogens (2). As an important part of the mucosal immune system, the intestinal mucosa faces great challenges by being constantly exposed to a large microbial community (3). In recent years, more and more studies have proved that the intestinal mucosa plays an important role in protecting the host against pathogen infection. For instance, Li et al (2012). characterized the role of the intestinal epithelial barrier in *Ictalurus punctatus* following *Edwardsiella ictaluri* challenge and identified 1,633 differentially expressed genes associated with immune activation and inflammatory responses (4). Moreover, TLR5 and the downstream MyD88-dependent signaling pathway were triggered in the intestine of *Danio rerio* after injection with a live attenuated *Vibrio anguillarum* vaccine (5). In addition, the major histocompatibility complex (MHC) was induced in the intestine of *Paralichthys olivaceus* after *E. tarda* infection (6). The above studies proved that the intestinal mucosal immune system plays a vital role in the immune responses against infection in teleost fishes. The exploration of the key immune genes and signaling pathways is needed to further characterize its molecular regulatory mechanism.

Increasing evidences have demonstrated that non-coding RNAs (ncRNAs), including long non-coding RNA (lncRNAs), microRNAs (miRNAs), and circular RNAs (circRNAs), are involved in the interactions between pathogens and their hosts (7–10). For example, large intergenic noncoding RNA (lincRNA) in *Oncorhynchus mykiss* was associated with immune response, which coexpressed with immune related genes such as integrin, Rab20, MHC class I genes, genes in PI3K or mTOR pathway, and genes in T cell receptor signaling pathway (11). A very recent report identified a novel lncRNA (*SETD3-OT*) in turbot with potential functions in regulating cell cycle and cell apoptosis, immune cell development, and immune response against infections (12). Most of the ncRNA studies have been focused on miRNAs in teleost. MiRNAs are small ncRNAs that play important roles in gene regulation at the posttranscriptional level by inhibiting mRNA translation or inducing mRNA degradation (13). Challenge studies performed in teleost fish have identified differentially expressed miRNAs that are associated with immune response genes and signaling pathways (14–22). For example, high-throughput sequencing and microarray analyses were used by Gong et al. (2015) to investigate the roles of miRNA such as miR-142-5p, miR-223, and miR-181a of *Cynoglossus semilaevis* in response to *V. anguillarum* infection (14). Similarly, high-throughput sequencing was also used to identify miRNAs that related with air-breathing organs in fish (15), with immune genes and associated signaling pathways after lipopolysaccharide (LPS) stimulation (16). Meanwhile, target genes of DE-miRNAs in common carp were enriched in focal adhesion, extracellular matrix (ECM)-receptor interaction, erythroblastic leukemia viral oncogene homolog (ErbB) signaling pathway, regulation of actin cytoskeleton, and adherent junction signal pathways (17). Based on the expression profiles of miRNAs, Liao et al. (2017) found regulationary functions of miRNAs in digestive and immune-related organs: gill, intestine, and hepatic caecum (18). Meanwhile, IL-1 receptor-associated kinase 4 (IRAK4) was identified and validated, which can be targeted by miR-203, thus inhibiting the activation of nuclear factor κB (NF-κB) signaling pathway (19). Besides, research found that miRNAs can regulate the toll-like receptor signaling pathways in teleost fish (20).

In addition to lncRNAs and miRNAs, circRNAs are a new category of ncRNA, which can be used as miRNA molecular sponges to influence the expression of miRNA, thereby affecting the synthesis of downstream target genes and signaling pathways (23). Because of their stable closed-loop structures, circRNAs could be perfect molecular markers for studying many diseases (24, 25). More importantly, recent studies have demonstrated that circRNAs have substantial effects on host-pathogen interactions in teleost fish. For example, the expression profiles of circRNAs in grass carp (*Ctenopharyngodon idella*) in response to grass carp reovirus infection were investigated, and 41 differentially expressed circRNAs were identified, which can bind to 72 miRNAs that may be associated with immune responses, blood coagulation, hemostasis, and complement and coagulation cascades (26). Moreover, 62 differentially expressed circRNAs were found in *E. tarda* infected intestinal tissues in *P. olivaceus*, which may be correlated with the herpes simplex infection pathway and IgA production pathway (6). However, only a few studies have been performed on the mechanism of interactions between ncRNAs and coding RNAs in teleost fishes, especially in the process of preventing pathogen infection in the intestine (6, 27). Moreover, there were no systematic study of the circRNAs, miRNAs, and mRNAs and the regulatory networks of competing endogenous RNAs (ceRNA) in *S. schlegelii* after pathogen infections.

*S. schlegelii* (black rockfish), one of the most popular and economically important aquaculture species, has a long-standing culture history in East Asian countries such as Japan, Korea, and China (28). With the expansion of the cultivation scale, numerous large outbreaks of bacterial and viral diseases have become major bottlenecks restricting its industry (29–31). Therefore, studies on black rockfish immune-related genes can expand our understanding of ncRNA that related to immune response and regulatory mechanisms, and are also helpful to guide the prevention and control of its diseases. Previous studies have investigated several immune-related genes, such as high mobility group box 1, c-type lectin, galectin-8, cathepsin K, chemokine ligand 25, and melanocortin-4, in black rockfish in response to pathogen stimulation (32–37). However, no systematic report on the interactions of ncRNAs and coding RNAs in *S. schlegelii* during infection has been performed.

In this study, we aimed to uncover the global landscape of mRNAs, circRNAs, and miRNAs in response to *E. tarda* infection at different time points (0, 2, 6, 12, and 24 h) to construct their regulatory networks. This study would not only provide novel insights into the roles of circRNAs, miRNAs, mRNAs, and relevant regulatory networks during pathogen infections but also broaden our understanding of the immune responses and regulatory mechanisms in the intestine of *S. schlegelii.*

## Materials and Methods

### Sample Collection and Bacterial Infection

The experimental adult *S. schlegelii* were obtained from a local fish farm in Qingdao, Shandong Province. And the experimental protocols were approved by the Committee on the Ethics of Animal Experiments of Qingdao Agricultural University IACUC (Institutional Animal Care and Use Committee). In this study, healthy black rockfish with an average length of 15 ± 2 cm were selected for following experiments. Then, these fish were acclimatized in the laboratory in a flow-through system filtered with seawater at 22 ± 1°C for a week before conducting bacterial infection experiments. Thereafter, a pre-challenge for *S. schlegelii* was performed, and *E. tarda* was isolated from the symptomatic fish. Subsequently, the isolated and confirmed *E. tarda* was cultured in LB medium at 28°C overnight at 180 rpm/min. The fish immersed in sterilized media were defined as the control (CON). At the same time, the fish in the experimental groups were immersed in *E. tarda* at a final concentration of 1 × 10^7^ CFU/ml for 4 h and then transferred into culture conditions. Subsequently, intestinal tissues from the *E. tarda*–infected groups were separately collected at different time points (2, 6, 12, and 24 h), and designated as EI2H, EI6H, EI12H, and EI24H, respectively. Each group had three replicates, and each replicate included 6 random individuals.

### Histopathological Examination

To observe the histopathological changes in intestinal tissues between control and *E. tarda*–infected *S. schlegelii*, intestines from 15 fish were isolated and fixed in 4% paraformaldehyde, dehydrated, and embedded in paraffin. Then, the embedded tissues were sectioned and stained with hematoxylin and eosin (H&E) according to a standard protocol reported by De Vico et al. for histological analysis (38).

### Total RNA Extraction and Quality Control

Total RNA from 15 samples was extracted using TRIzol Reagent (Invitrogen, Carlsbad, CA, USA) and were further treated with RNase-free DNase I to remove DNA (TIANGEN, Beijing, China). The concentration, purity, and integrity of the RNA samples were assessed using a NanoPhotometer spectrophotometer (IMPLEN, CA, USA) and Bioanalyzer 2100 system (Agilent Technologies, CA, USA). High-quality RNAs were used for the construction of the sequencing libraries.

### RNAs Libraries Construction, Sequencing, and Data Processing

For both mRNA and circRNA library construction, 5 μg of RNA per sample was used as the input. For mRNA library construction, ribosomal RNA (rRNA) was removed from the total RNA using an Epicenter Ribo-Zero rRNA Removal Kit (Illumina, USA). For circRNA library construction, 40 U RNase R was added to the rRNA removal system and incubated at 37°C for 3 h to remove linear RNA. Subsequently, the purified RNAs were fragmented to 150–200 bp and used to construct the sequencing library using Illumina TruSeq RNA Sample Preparation Kit (Illumina, San Diego, USA) according to the manufacturer’s recommendations with different indices. Before sequencing, the quality of the mRNA library was detected using the Agilent Bioanalyzer 2100 system. After sequencing on an Illumina Hiseq 2500 platform, the raw data generated were filtered to remove low-quality reads, adapter sequences, and reads containing Ns. Then, the clean data were aligned to the assembled genome of *S. schlegelii* using TopHat v2.0.12 (39). For circRNA identification, we used the following criteria for circRNA identification: 1) both ends of splice sites must be GU/AG; 2) mismatch ≤ 2; and 3) back-spliced junction reads ≥ 1. The two splice sites must not be more than 100 kb apart on the genome according to the structural characteristics of circRNA. To explore expression patterns, mRNA or circRNA expression was calculated as reads per kilobase per million reads (RPKM). The differential expression analysis of mRNAs or circRNAs was performed using the DESeq2 R package (1.10.1) (40). The criterion of adjusted *P*-value < 0.05 was used to identify differentially expressed RNAs. Data are available from Dryad at: https://doi.org/10.5061/dryad.7pvmcvdrp.

For miRNA library construction, a total of 5 μg of total RNA per sample was used as input to construct a small RNA library using the NEBNext^®^ Multiplex Small RNA Library Prep Set for Illumina^®^ (NEB, USA) according to the manufacturer’s recommendations, and index codes were added to each sample. Briefly, the 5’ ends and 3’ adapters were specifically ligated to the 5 and 3’ ends of miRNA, siRNA, and piRNA, respectively. After ligation, the first-strand cDNA was synthesized using M-MuLV Reverse Transcriptase (RNase H) and then amplified using LongAmp Taq 2X Master Mix, SR Primer for Illumina, and index primer. Subsequently, the PCR products ranged from 140 to 160 bp were recovered from an 8% polyacrylamide gel for the final miRNA library construction. Similarly, library quality was detected on the Agilent Bioanalyzer 2100 system using DNA High Sensitivity Chips. After sequencing and filtration of raw data, clean reads were obtained. Then, small RNA tags were mapped onto the assembled genome of *S. schlegelii* using Bowtie (41). The mapped small RNA tags were screened in the miRBase 20.0 database to identify known miRNAs. In addition, mirdeep2 and srna-tools-cli were used to obtain potential miRNA and draw secondary structures (42). For novel miRNA prediction, miREvo (43) and mirdeep2 (42) were integrated to predict the novel miRNAs based on the characteristics of the hairpin structure of the miRNA precursor. The counts and base bias at the first position of all known and novel miRNAs were calculated using perl scripts. Two databases, miFam.dat (http://www.mirbase.org/ftp.shtml) and Rfam (http://rfam.sanger.ac.uk/search/) were explored for the occurrence of miRNA families. For function analysis of miRNAs, miRanda was used to predict their target genes (44). MiRNA expression was estimated using TPM (transcript per million) (45). Significantly, differentially expressed miRNAs were identified using the threshold: *P*-value < 0.05. To further understand the functions of these significantly differentially expressed (DE) circRNA, mRNA, and miRNAs, Gene Ontology (GO) and Kyoto Encyclopedia of Genes and Genomes (KEGG) enrichment analyses were performed using the GOseq R package and KOBAS, respectively (46, 47).

### Co-Expression Network Analysis

The circRNA-miRNA, miRNA-mRNA, and circRNA-miRNA-mRNA networks were developed based on possible functional relationships between DE-circRNAs, DE-miRNAs, and DE-mRNAs. The candidate miRNA-circRNA relationships were predicted using miRanda (44) by thresholds: total sore ≥ 140; total energy < 17 kmol). Similarly, miRanda (44) was used to predict the target DE-mRNAs of DE-miRNAs. Then, a miRNA-mRNA regulation network was constructed. In the constructed miRNA-mRNA network, these confirmed miRNAs were selected as candidates to predict the corresponding circRNA-miRNA pairs. Finally, the co-expression relationships among circRNAs, mRNAs, and miRNAs were selected to establish the regulatory network using Cytoscape (v3.4.0) (48).

### Validation of the Differential Expression of circRNAs, miRNAs, and mRNAs

To validate the differential expression of circRNAs, miRNAs, and mRNAs, the samples were prepared using the same method mentioned in the RNA library construction and sequencing section. To validate the expression patterns of the differentially expressed circRNAs and mRNAs, total RNAs of the *E. tarda* infected *S. schlegelii* and control groups were extracted using TRIzol reagent (Invitrogen, Carlsbad, CA, USA), and then reverse transcribed into cDNA using a PrimeScript™ RT reagent Kit (Takara, Otsu, Japan) according to the manufacturer’s instructions. Hereafter, ribosomal protein L17 *(RPL17)* was used as an internal control for the normalization of gene expression (49). Primers for the 6 DE-mRNAs and 6 DE-circRNAs for qRT-PCR analysis were designed using PrimerQuest (https://sg.idtdna.com/PrimerQuest/Home). For circRNA primers, the primers were designed according to the sequences that span the circRNA backsplice junctions. For miRNA primers, we designed the forward primers according to the standards mentioned in the miRcute miRNA isolation kit (Tiangen Biotech). Subsequently, the expression levels of these genes were analyzed using a CFX96 real-time PCR detection system (Bio-Rad Laboratories, Hercules, CA, USA). The reaction systems contained 0.4 μl of the diluted template cDNA, 0.2 μl of each primer, 4.2 μl of SYBR^®^ Premix Ex TaqTM II (TliRNaseH Plus), and 5.0 μl of RNA-free water. The reaction mixtures were pre-denatured for 5 min at 95°C, followed by 35 cycles of 95°C for 5 s, 56°C for 30 s, and 72°C for 30 s, and then up to 95°C at a rate of 0.1°C/s increments for melting curve analysis. The differentially expressed miRNAs were also confirmed using qRT-PCR. First, small RNAs (<200 nt) were extracted with the miRcute miRNA isolation kit (Tiangen Biotech) and amplified using the miRcute miRNA qPCR detection kit (Tiangen Biotech) under the following PCR conditions: 95°C for 15 min, followed by 40 cycles of two steps (95°C for 5 s and 60°C for 30 s). Next, 5S rRNA was selected to normalize the relative expression levels of 6 DE-miRNAs. Finally, the 2^−ΔΔCt^ method was used to calculate the relative fold changes (50). The data are shown as the mean ± SE of three replicates. All the primers used in this study are listed in Supporting **Table S1**.

## Results

### Histopathological Analysis

**Figure 1:**
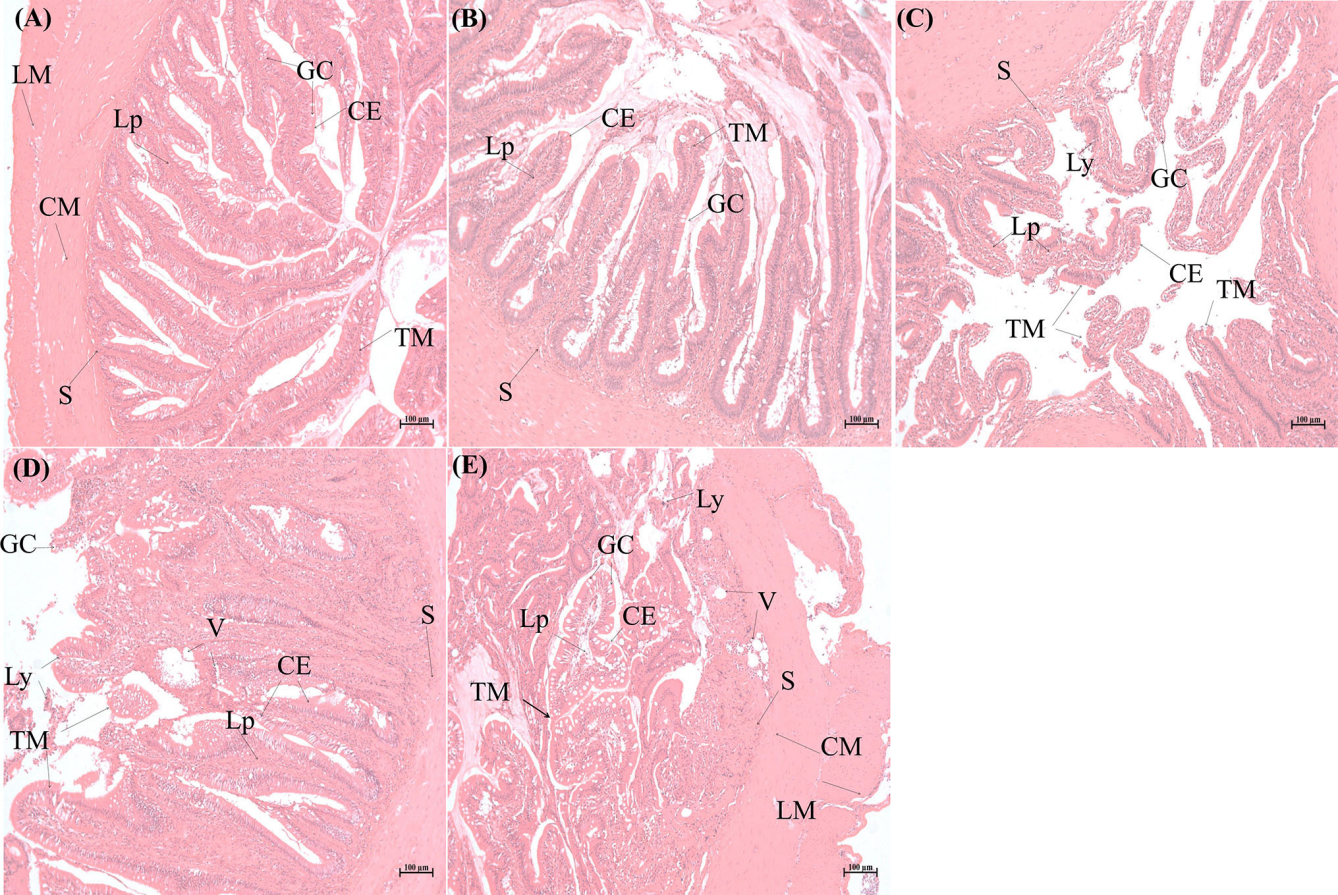
Histopathological analysis of intestinal tissues of *Sebates schlegelii* following *Edwardsiella tarda* challenge. **(A)** is the representative microstructure of healthy intestine of the control. **(B–E)** represent the microstructures of intestines 2, 6, 12, and 24 h after *E. tarda* infection, respectively. CE, columnar epithelial cell; CM, circular muscularis; GC, goblet cell; LM, longitudinal muscularis; Lp, lamina propria; Ly, lymphocytes; S, submucosa; TM, tunica mucosa; V, vacuole.

To understand the histopathological changes in intestinal tissues when exposed to pathogenic bacteria, *E. tarda* was used to infect *S. schlegelii* and the infected intestinal tissues were dissected for histopathological analysis. The intestinal tissues from the control group and different infection time points (2, 6, 12, and 24 h) were observed for morphological structures. Light microscopic examination of the normal intestine showed that the mucosal folds, submucosa, circular muscular layer (muscularis), serosa, lamina propria, goblet cells, and epithelium were visible and well arranged (**Figure 1A**). As the infection time progressed, the integrity of the intestinal mucosa structure changed and further microscopic examination showed hyperplasia of the mucosa, thickening of the lamina propria, epithelial cell shedding, mucosal fold breakage, increase in goblet cells, and changes in autolysis and necrosis. At 2 h post-infection, the structure of the intestinal tissue was complete, goblet cells increased in number, and some wandering cells and a few vacuoles interlined the epithelium (**Figure 1B**). After 6 h of infection, the goblet cells continued to increase, the width of the mucosa and lamina propria increased, and some epithelium structure started to disassemble, and more apical opening of the goblet cells was observed. In addition, more lymphocytes were detected in the vacuoles close to the submucosa (**Figure 1C**). Distinct autolysis was observed on the surface of the mucosa and epithelial cells. Autolytic changes also included intestinal necrosis. Some of the lamina epithelial cells were pinched off, clustered, and packed. Significantly large vacuoles were observed, which might be entangled with pinocytotic vesicles, and the intestinal structures are destroyed (**Figure 1D**). When dissecting the intestinal tissues, we found that it had lost its elasticity and broke easily when pulled in some portions (data not shown). More goblet cells were seen, some budded off, and the lamina propria structure was further loosened. Numerous lymphoid cells were scattered in the lamina propria and the vacuole of the cytoplasm (**Figure 1E**). The intestinal villi was deformed and shed, and the folded mucosa was destroyed (**Figure 1E**). Our results indicated that the destruction of *E. tarda* to intestinal tissues of *S. schlegelii* is a time-dependent manner.

### Identification, Quantification, and Differential Expression Analysis of circRNAs, miRNAs, and mRNAs

#### Identification of circRNAs in Response to *E. tarda* Infection in *S. schlegelii*

**Figure 2:**
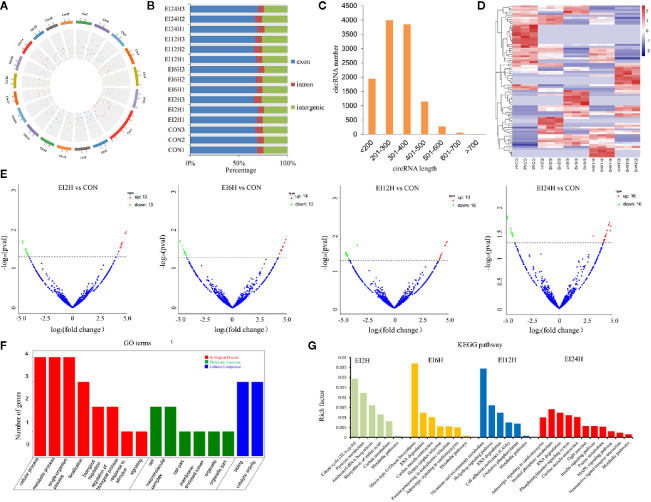
circRNA expression overview in the intestinal of *Sebates schlegelii* following *E. tarda* challenge. **(A, B)** Genome location of circRNAs. **(C)** circRNAs length distribution. **(D)** Heatmap of DE circRNAs among control and infected groups. **(E)** Volcano Plots of DE circRNAs among control and infected groups. Red blocks represent up-regulated circRNAs and green blocks represent down-regulated circRNAs. **(F)** Go term analysis of DE circRNAs. **(G)** KEGG analysis of DE circRNAs.

To understand the roles of circRNAs in response to *E. tarda* infection in the intestine of *S. schlegelii*, we performed circRNA sequencing using the rRNA-depleted samples of non-infected (CON) and infected samples at different time points (2, 6, 12, and 24 h). The results showed that RNA purity, concentration, and the amount of RNA met the requirements for circRNA database construction. As shown in **Table S2**, there were approximately 47.72, 56.82, 50.35, 42.12, and 42.48 million clean reads in the CON, EI2H, EI6H, EI12H, and EI24H groups, respectively. The Q20 values of these data were higher than 97%, and the average GC content was 54.17%. Among them, 74.08% of the reads were aligned to the genome. Then, the clean reads were used for circRNA identification. In total, we identified 2,629 circRNAs in *S. schlegelii*, which were widely distributed on 20 chromosomes (**Figure 2A** and Dataset 1). Exon cyclized RNA is formed by a splice donor downstream of the exon that is ligated to a splice acceptor upstream of the exon. Since the order of the exons has been rearranged, the normal linear alignment cannot obtain the ring-spliced reads. Therefore, we obtained backspliced ​​junction reads. The valid data can be aligned to the reference genome and defined as exons, introns, and intergenic regions. The results showed that 68.01% of circRNAs were composed of exons, while 6.69% and 25.30% were located in the intronic and intergenic regions, respectively (**Figure 2B**). In addition, we found that the size of most circRNAs ranged from 200 to 400 bp (**Figure 2C**). To explore the expression patterns of these circRNAs, a hierarchical cluster analysis of 87 differentially expressed circRNAs (DE-circRNAs) was performed among the CON, EI2H, EI6H, EI12H, and EI24H groups, which classified the expression patterns of the uninfected and *E. tarda* infected samples into different clusters (**Figure 2D**). There were two, three, one, and four clusters that showed upregulation in the *E. tarda* infected groups at 2, 6, 12, and 24 h post-infection when compared with control group, respectively. Furthermore, the differences in circRNA expression patterns among different time points and controls were analyzed (**Figure 2E**). A total of 26 DE-circRNAs were observed at early infection time points when compared to the control. Among them, half were upregulated and half were downregulated. At 6 h post-infection, 14 and 13 circRNAs were found to be upregulated during *E. tarda* infection. Moreover, 13 circRNAs were found to be significantly upregulated and 16 circRNAs were significantly downregulated at 12 h post-infection relative to the control groups. In addition, 32 DE-circRNAs were detected in the 24 h post-infection groups with half upregulated and the other half downregulated.

To elucidate the biological function of circRNAs in *S. schlegelii* after *E. tarda* infection, we performed GO and KEGG functional analysis of these DE-circRNAs. GO is an internationally standardized gene function classification system that provides a dynamically controlled vocabulary to fully describe the properties of genes and gene products in an organism. The 125 core GO terms of four time points post-infection were extracted (**Figure 2F** and **Figure S1**), which can be classified into biological processes, cellular components, and molecular functions. Regarding molecular function, GO terms such as cellular process, metabolic process, single-organism process, localization, biological regulation, regulation of the biological process, and response to stimulus were enriched. In the cellular component, GO terms such as cell, macromolecular complex, cell part, organelle, and organelle part were functionally enriched. Meanwhile, binding and catalytic activity were the most important molecular functions. To further focus on the function of these DE-circRNAs, KEGG pathways were analyzed and enriched. The results showed 6, 7, 6, and 11 circRNAs in the four groups (EI2H, EI6H, EI12H, and EI24H), which were involved in 23 KEGG metabolic pathways, such as tight junction and MAPK signaling pathways (**Figure 2G**).

#### Identification of DE miRNAs Between *E. tarda*–Infected and Control Groups

**Figure 3:**
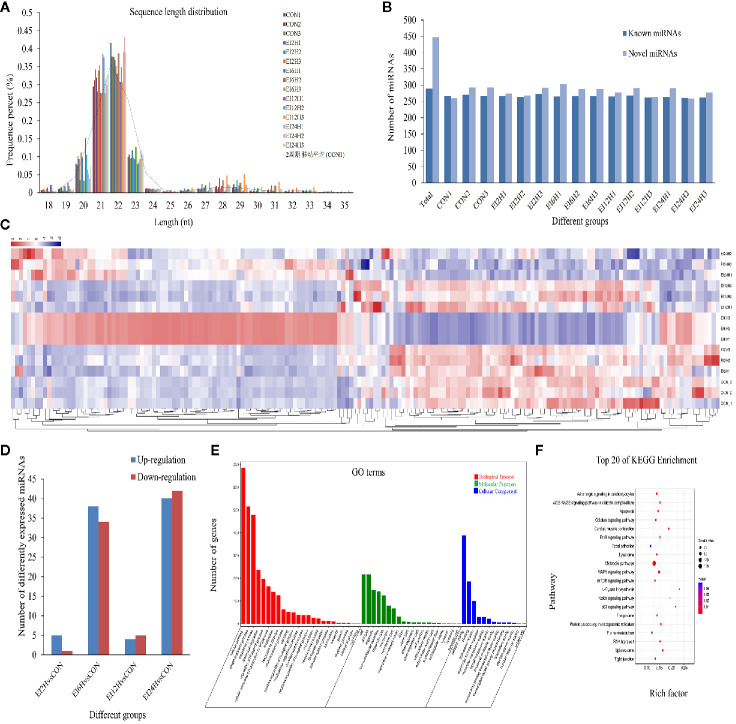
The miRNA expression overview in the intestine of *Sebates schlegelii* following *E. tarda* challenge. **(A)** The length distribution of identified miRNAs. **(B)** Number of known and novel miRNAs. **(C)** Heatmap of DE miRNAs among control and infected groups. **(D)** DE miRNAs among control and infected groups. **(E)** Go term analysis of DE miRNAs. **(F)** KEGG analysis of DE miRNAs.

To explore which miRNAs showed differential expression patterns in response to *E. tarda* infection, we identified miRNAs in *S. schlegelii* and investigated the expression patterns of these miRNAs at different time points (0, 2, 6, 12, and 24 h). After removing the low-quality raw reads and mRNA, Rfam, and Repbase mappable reads, the average bases of CON, EI2H, EI6H, EI12H, and EI24H samples were 0.59, 0.58, 0.60, 0.56, and 0.54 Gb, respectively (**Table S3**). These reads were used for further miRNA identification and function analysis. We found that 68.78%, 66.40%, 63.86%, 67.39%, and 67.40% of small RNA reads were mapped onto the *S. schlegelii* genome (**Table S4**). As shown in **Figure 3A**, the length of these sRNAs ranged from 18 to 35 nt, with 22 nt as the dominant one, followed by 21 and 23 nt, respectively (**Figure 3A**). The first nucleotide bias analysis of miRNAs in *S. schlegelii* showed that the first residues of the 21, 22, and 23 nt miRNAs were predominantly uridine (U) (**Figure S2**).

A total of 736 miRNAs were identified, including 289 conserved miRNAs and 447 novel miRNAs (**Figure 3B** and Dataset 2). Sequencing analysis revealed that 177 miRNAs exhibited significant variation after *E. tarda* infection. Their transcriptional patterns along the time course of infection showed expression patterns at 2 and 12 h, which were similar to those of the control group. Moreover, we found that the miRNAs were most responsive at 6 h post-infection. Besides, some miRNAs were induced after a long period of infection (**Figure 3C**). To further clarify the miRNA response mechanism, we compared the differences between the miRNAs at different time points after infection and the miRNA in the control group. The results showed that 6, 72, 9, and 82 DE-miRNAs exhibited significant variation in H2 vs. H0, H6 vs. H0, H12 vs. H0, and H24 vs. H0 comparisons, respectively (**Figure 3D**). To further explore the function of these DE-miRNAs, their target genes and functions were statistically analyzed. The results showed that these DE-miRNAs target 3,750 genes. GO analysis showed that these target genes were mainly enriched in the 3,245 GO term process (**Figure 3E**). Analysis of the KEGG metabolic pathway showed that these genes were involved in metabolic pathways (158), MAPK signaling pathway (47), calcium signaling pathway (35), protein processing in the endoplasmic reticulum (28), focal adhesion (28), tight junction (24), and apoptosis (25) (**Figure 3F**).

#### Identification of DE mRNAs Between *E. tarda*–Infected and Control Groups

**Figure 4:**
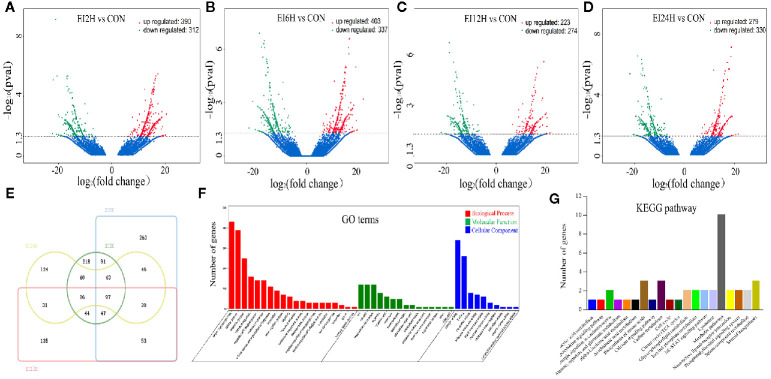
Analysis of mRNA sequencing data in the intestine of *Sebates schlegelii* following *E. tarda* challenge. **(A–D)** Volcano plots were drawn to visualize the standardized expression of mRNAs between the infected and control groups. The red and green points represent differentially expressed mRNAs with statistical significance (P < 0.05). **(E)** Veen diagram of mRNAs. **(F)** Go term analysis of DE mRNAs. **(G)** KEGG analysis of DE mRNAs.

To identify the different expression levels of mRNAs in the *E. tarda* infected groups and control group, 15 mRNA libraries were constructed. Finally, 702, 740, 497, and 609 genes were identified as DEGs in EI2H, EI6H, EI12H, and EI24H libraries, respectively, compared with CON (**Figures 4A–D**). A total of core 97 genes showed different expression levels at all the infected time points (**Figure 4E**). Furthermore, GO analysis and KEGG pathway analysis were used to examine the functions of these DE mRNAs. GO analysis was conducted with the DE mRNAs in *S. schlegelii* using the Blast2GO program to classify their biological functions. In detail, 2,011 DE mRNAs were successfully assigned to GO terms with 1,070 functional terms (**Figure 4F**). Among them, 57.03%, 24.05%, and 18.92% DE miRNAs were assigned to the biological process, molecular function, and cellular component categories, respectively. To further elucidate the physiological implications and interactions of the DE mRNAs identified in our sequencing analysis, we BLASTed the DE mRNAs against referenced canonical pathways in the KEGG database using BLASTx with an E-value cutoff of 1e-5. KEGG analysis showed that the enriched pathways were mainly involved in metabolic pathways, biosynthesis of amino acids, and carbon metabolism (**Figure 4G**).

### Co-Expression Network Construction

#### Construction of the Potential circRNA-miRNA Co-Expression Network

CircRNA molecules are rich in miRNA binding sites and acting as miRNA sponges in cells, thereby abolishing the inhibition of miRNAs on their target genes and increasing the expression levels of target genes, which was defined as a competitive endogenous RNA (ceRNA) mechanism (51). Therefore, an integrative analysis of the interplay between circRNAs and their target miRNAs was performed to elucidate their functional connections. Taken together, we identified 148 miRNAs that were bound to 156 circRNAs with different expression levels and generated 624 circRNA and miRNA pairs (**Table S5**). In detail, novel_circ_0004195 may function as ceRNAs and sequester novel_530 to relieve its binding and targeting of 124 mRNAs (including cytokine receptor-like factor, the inhibitor of kappa B (*IκB*), cohesin, and transcription factor) (**Table S6**). We also found that five circRNAs (novel_circ_0001019, novel_circ_0002395, novel_circ_0003142, novel_circ_0003744, and novel_circ_0003853) have binding sites for novel_663, which targets the G-protein coupled receptor, E3 ubiquitin-protein ligase, and myomaker. In addition, the relationship between dre-miR-203a-3p and novel_circ_0001819, dre-miR-150, novel_circ_0003210, and novel_circ_0003372 were also predicted. The target genes of the two miRNAs are related to transmembrane protein 65, MAP kinase-activated protein kinase, and tetraspanin. Taken together, these circRNAs/miRNAs could play important roles in host defense when organisms are infected by pathogenic bacteria.

#### Construction of the Potential miRNA-mRNA Co-Expression Network

In the present study, the regulatory networks of DE-miRNAs and their corresponding target mRNAs were constructed and investigated using the MiRanda software. In total, 4,545 mRNAs were found to be targeted by 79 DE-miRNAs (**Table S6**). Among them, 7 (8.86%) miRNAs were found to target only one mRNA, such as dre-miR-203a-3p, ccr-miR-142-3p, dre-miR-454b, and novel_264. However, most miRNAs could target more than one mRNA. For instance, novel_277, dre-miR-23b-5p, dre-miR-24b-3p, and novel_10 had 488, 484, 329, and 264 target mRNAs, respectively. In addition, many mRNAs were associated with more than one miRNA; for example, the immunoglobulin superfamily DCC subclass member 3 was targeted by ccr-miR-128 and dre-miR-128-3p. Methyltransferase-like protein 24 was targeted by ccr-miR-10d, dre-miR-10d-5p, and novel_49. The results indicate that complex miRNA-mRNA regulatory networks existed in the process of pathogen invasion and host defense.

#### Construction of the Potential circRNA-miRNA-mRNA Network

**Figure 5:**
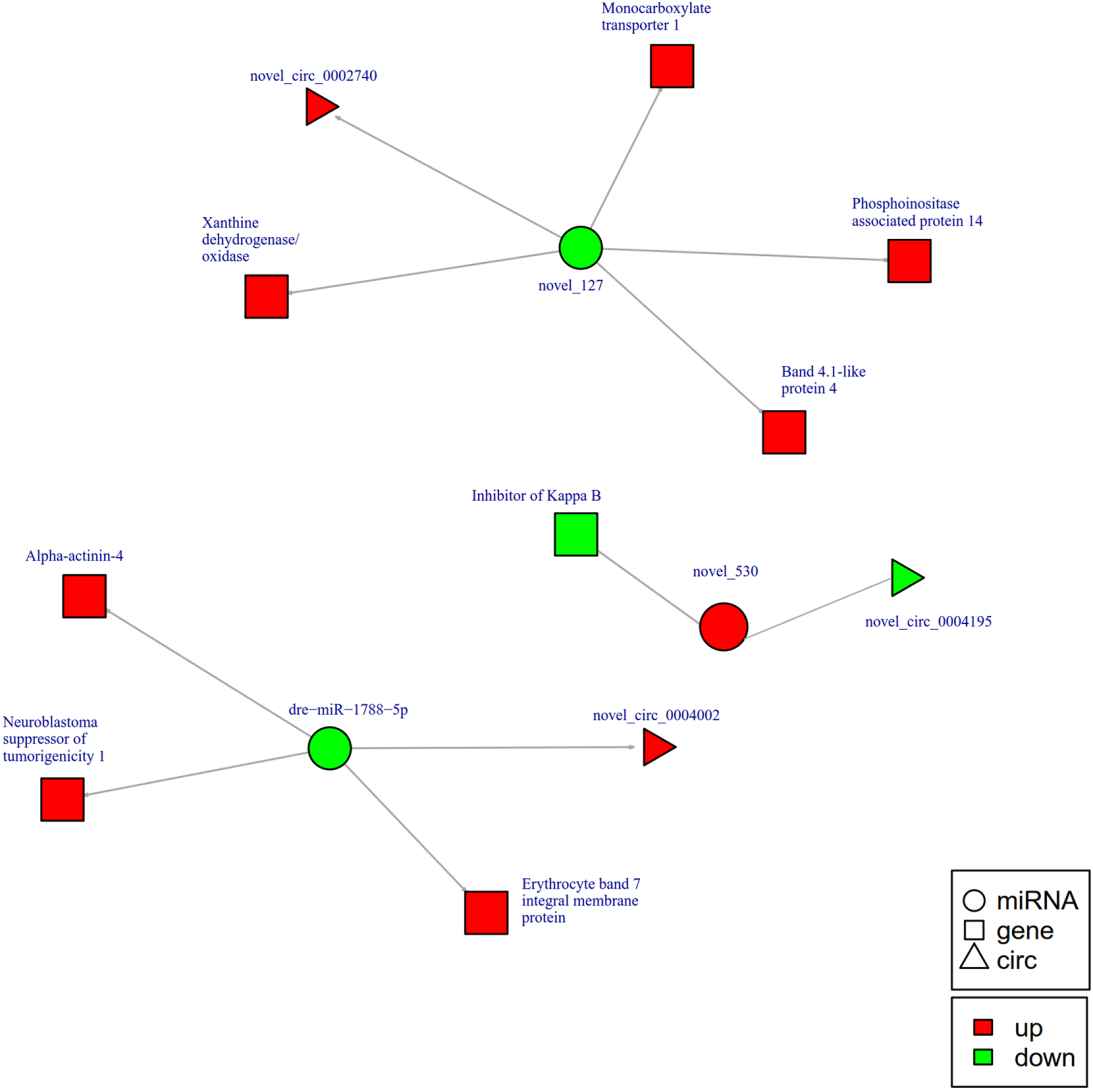
The circRNA-miRNA-mRNA interaction networks in the intestine of *Sebates schlegelii* fowllowing *E. tarda* infection at 2 h. Circles represent miRNA, triangles represent circRNA, and squares represent mRNA. Red and green shaded represent up-regulated and down-regulated RNAs, respectively.

**Figure 6:**
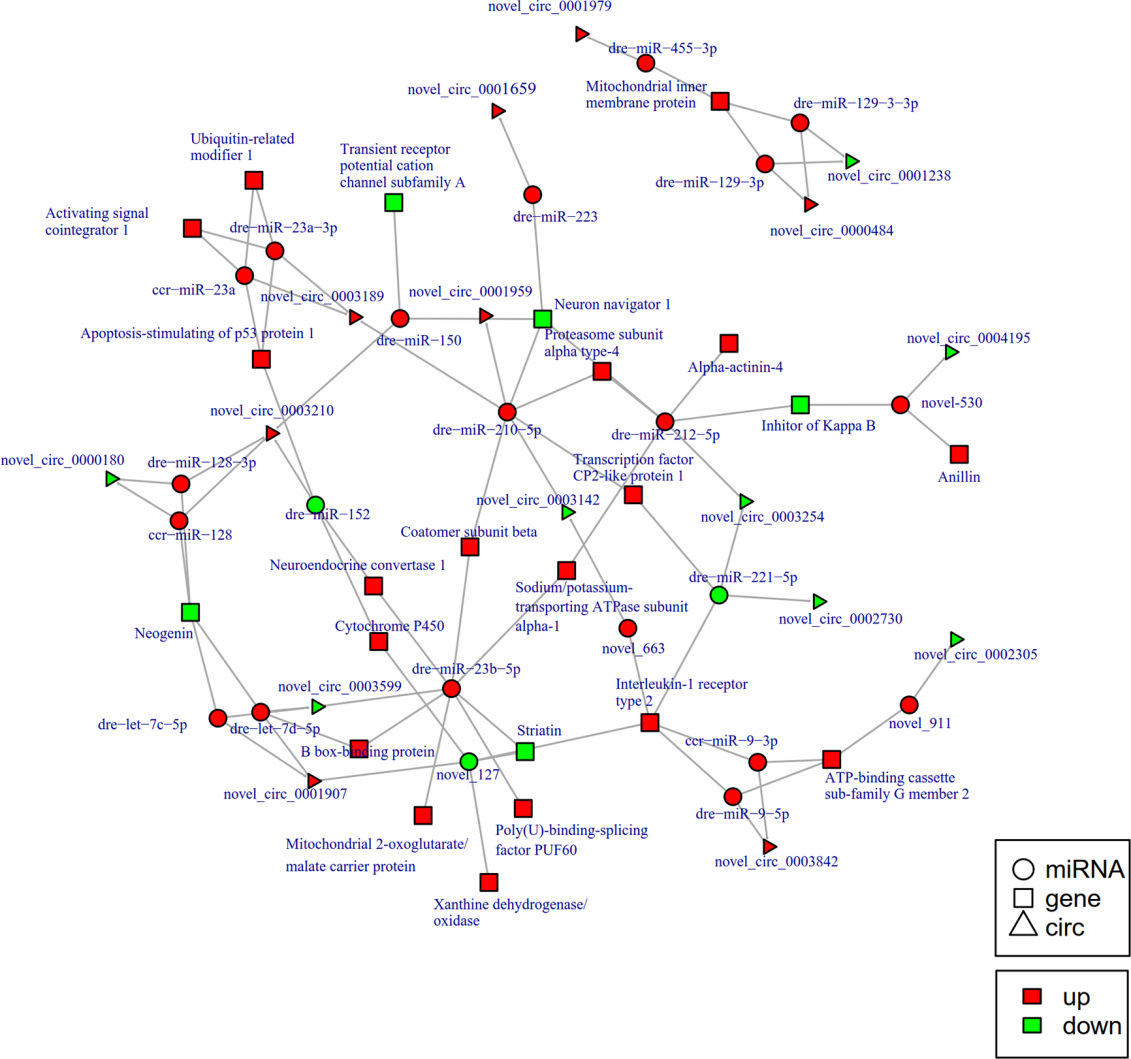
The circRNA-miRNA-mRNA interaction networks in the intestine of *Sebates schlegelii* fowllowing *E. tarda* infection at 6 h. Circles represent miRNA, triangles represent circRNA, and squares represent mRNA. Red and green shaded represent up-regulated and down-regulated RNAs, respectively.

**Figure 7:**
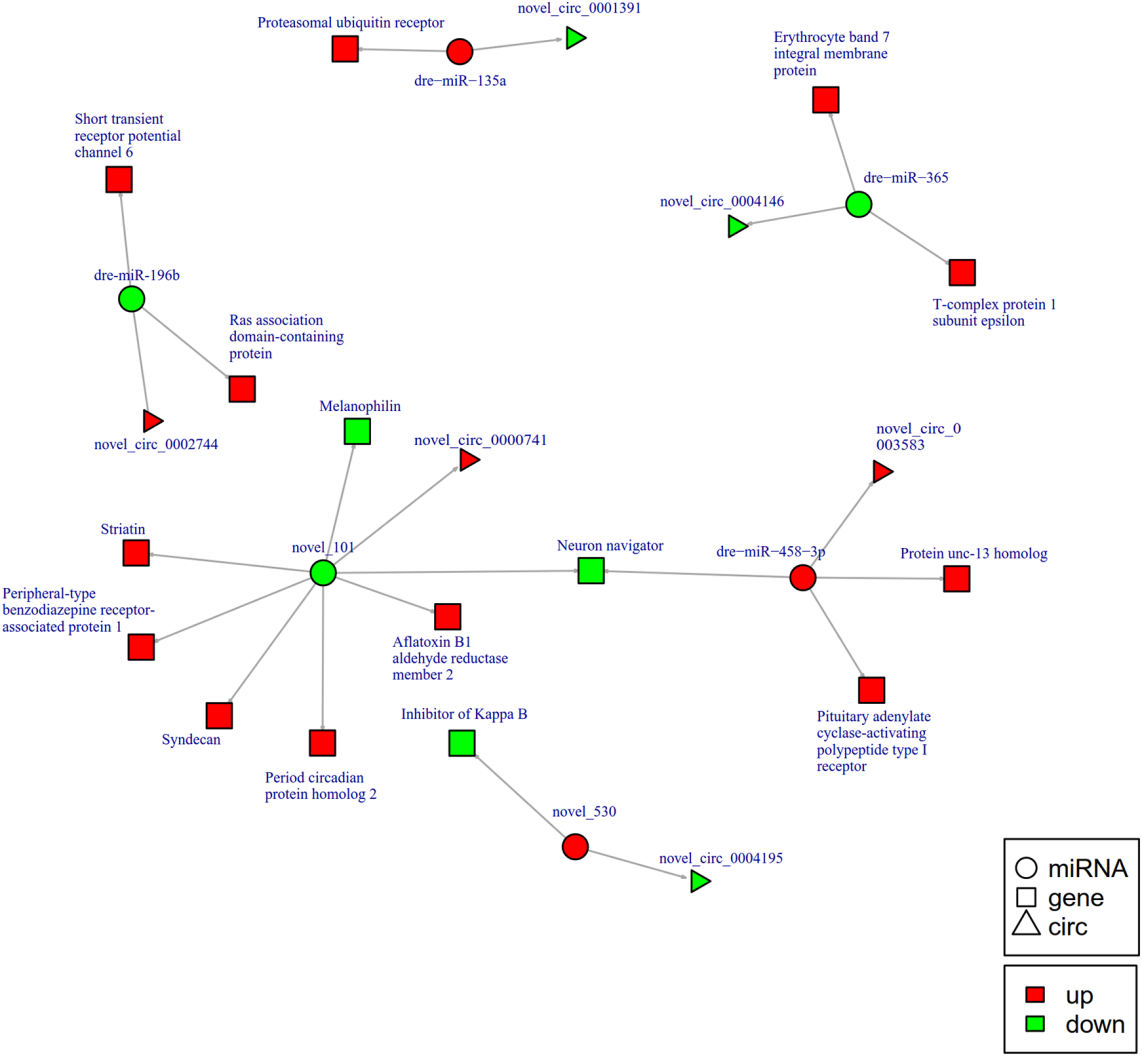
The circRNA-miRNA-mRNA interaction networks in the intestine of *Sebates schlegelii* fowllowing *E. tarda* infection at 12 h. Circles represent miRNA, triangles represent circRNA, and squares represent mRNA. Red and green shaded represent up-regulated and down-regulated RNAs, respectively.

**Figure 8:**
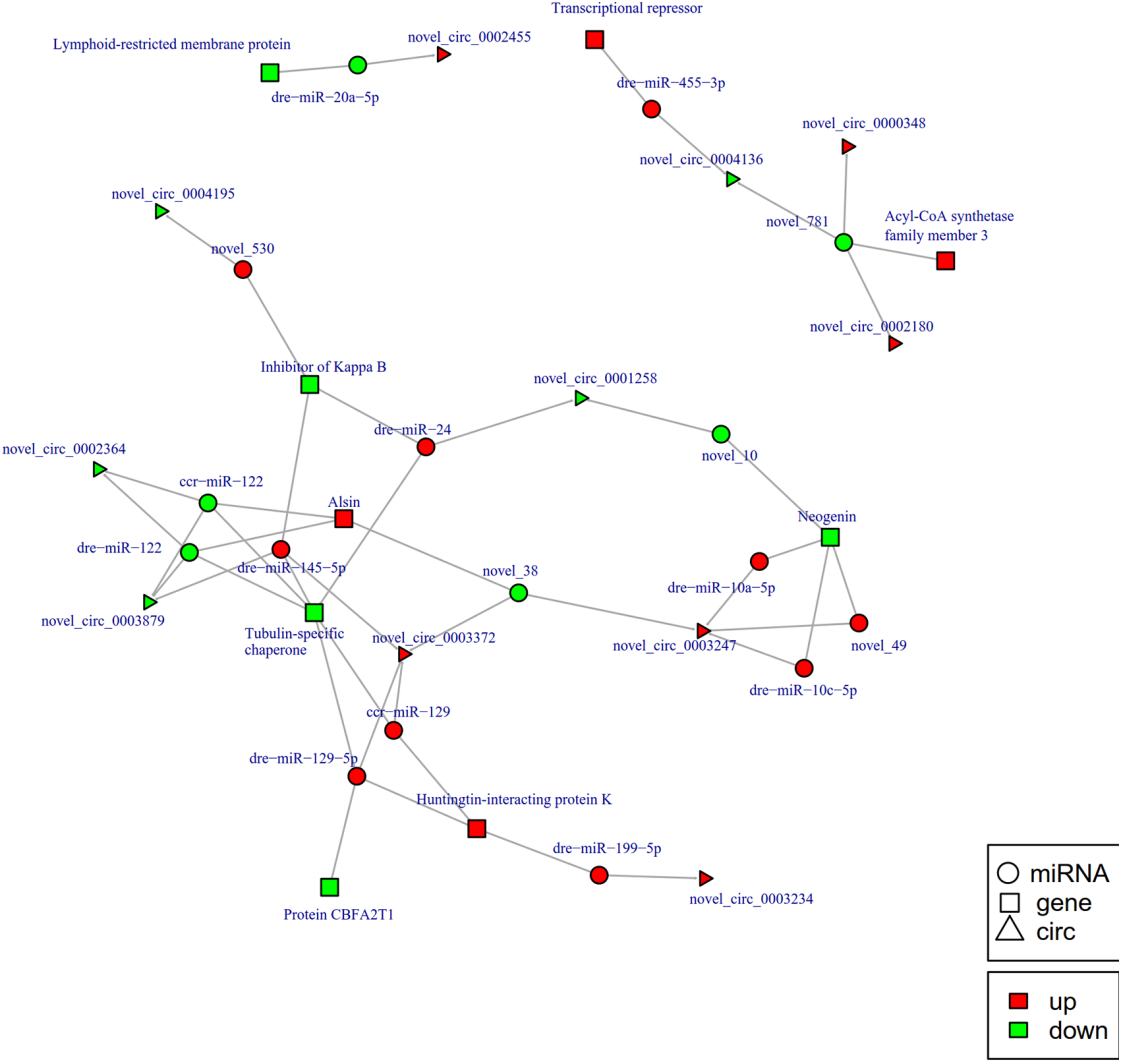
The circRNA-miRNA-mRNA interaction networks in the intestine of *Sebates schlegelii* fowllowing *E. tarda* infection at 24 h. Circles represent miRNA, triangles represent circRNA, and squares represent mRNA. Red and green shaded represent up-regulated and down-regulated RNAs, respectively.

To further explore the potential network of circRNAs, miRNAs, and mRNAs, we constructed circRNA-miRNA-mRNA co-expression networks based on the circRNA-miRNA and miRNA-mRNA results (**Figure 5**). The DEcircRNA-miRNA-mRNA networks suggested that 17 downregulated circRNAs bound to 22 miRNAs and 26 miRNA-targeted mRNAs, while 19 upregulated circRNAs were linked to 30 miRNAs and 37 miRNA-targeted mRNAs. In the network of circRNA-miRNA-mRNA 2 h post-infection, two upregulated circRNAs (novel_circ_0002740 and novel_circ_0004002) related to miRNA novel_127 and dre-miR-1788-5p, and the two miRNAs radiated to their target genes such as Interleukin-1 receptor type 2, a neuroblastoma suppressor of tumorigenicity 1. In contrast, the downregulated novel_circ_00004195 was connected to novel_530 and *IκB* (**Figure 5**). When constructing the potential circRNA-miRNA-mRNA network at 6 h post-infection, we found that at the center of the network, novel_circ_0001907 and novel_circ_0003210 were upregulated. We also found a network containing novel_circ_00004195 that was downregulated. The three circRNAs radiated to their respective predicted miRNAs, novel_127, dre-miR-152, and novel_530. Meanwhile, their related miRNAs were connected to their respective target mRNAs such as xanthine dehydrogenase/oxidase, interleukin-1 receptor type 2, cytochrome P450, apoptosis-stimulating of p53 protein 1, neuroendocrine convertase 1, inhibitor of kappa B (*IkB*), and anillin (**Figure 6**). According to our results, miRNAs including novel_530, dre-miR-101, and dre-miR-1966, radiated three circRNAs (novel_circ_00004195, novel_circ_00000741, and novel_circ_0002744) and their target genes (*IkB*, short transient receptor potential channel 6 and ras association domain-containing protein 2 and others) at 12 h (**Figure 7**). Furthermore, five co-expression networks for circRNAs regulating targeted miRNAs and miRNAs regulating targeted mRNAs were formed after 24 h of infection. In detail, miRNA_38 was linked by anlsin and was linked by novel_circ_0003372 and novel_circ_0003247. MiRNA novel_781 radiated to acyl-CoA synthetase family member 3, novel_circ_0000348, and novel_circ_0002180. MiRNA novel-530 radiated to novel_circ_0004195 and *IkB*. In addition, a network containing novel_circ_0002455/dre-miR-20a-5p/lymphoid-restricted membrane protein was also found at this time point (**Figure 8**). These circRNA-miRNA-mRNA networks can be selected as candidates for the following functional analysis.

### GO and KEGG Analysis of circRNA Co-Expression Genes

**Figure 9:**
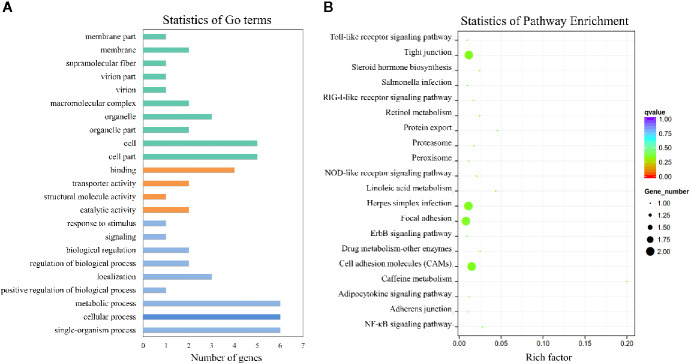
GO and KEGG analysis of the circRNA co-expression genes. **(A)** GO analysis of circRNA co-expression genes. **(B)** Statistics of KEGG pathways enrichment of circRNA co-expression genes. The colorful bar refers to the q-value of the respective signaling pathway. Size of the point refers to the number of genes within each pathway.

To better understand the mechanisms that responded to *E. tarda* infection, GO and KEGG enrichment analysis was performed to explore the function of circRNA co-expression genes. GO enrichment analysis revealed 23 terms, which are presented in **Figure 9A**. The results showed that the most enriched GO terms were strongly associated with single-organism process (GO: 0044699), cellular process (GO: 0009987), metabolic process (GO: 0008152), cell part (GO: 0044464), and cell (GO: 0005623). KEGG pathway analysis was conducted to characterize the targeted genes (**Figure 9B**), which were predicted to be related to herpes simplex infection, cell adhesion molecules (CAMs), focal adhesion, and tight junctions.

### Validation of the Differentially Expressed circRNAs, miRNAs, and mRNAs *via* qRT-PCR

**Figure 10:**
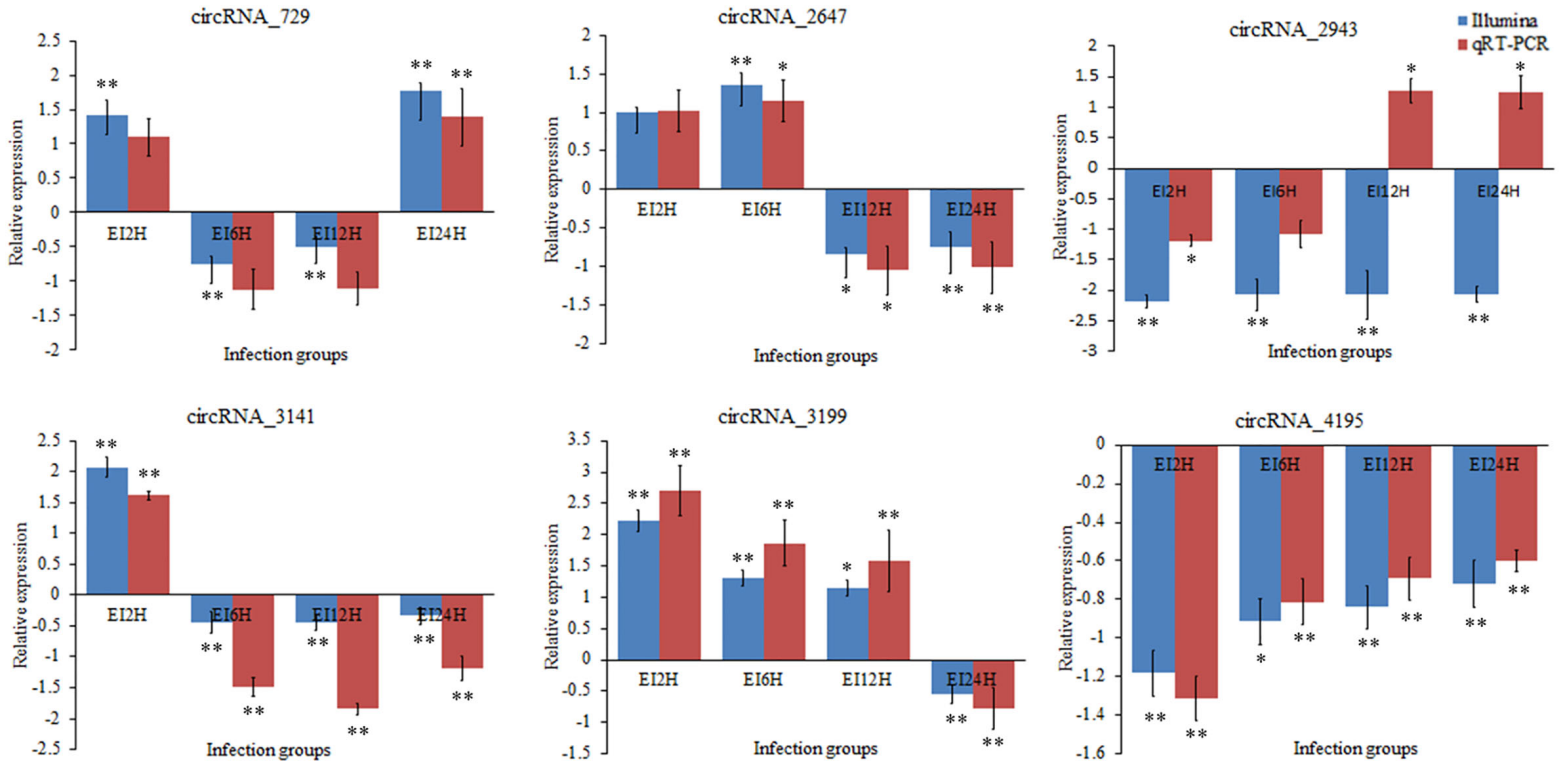
Validation of circRNAs by qRT-PCR analysis. The expression patterns of qRT-PCR were presented between control and, 2, 6, 12, and 24 h infection groups. The results showed the relative fold change and their mean ± standard error (SE) from triplicate experiments. ** indicates significance at the 0.01 level, * indicates significance at the 0.05 level.

To validate the expression levels of circRNAs, miRNAs, and mRNAs obtained by sequencing, we randomly selected six circRNAs (circRNA_729, circRNA_2647, circRNA_2943, circRNA_3141, circRNA_3199, and circRNA_4195), six miRNAs (novel_530, novel_186, novel_663, dre-miR-150, dre-miR-210-5p, and dre-miR-455-3p), and six mRNAs [insulin receptor, dachshund homolog, protein Mpv17, aquaporin, NLRC3, and inhibitor of kappa B (*IkB*)] and measured their expression levels after *E. tarda* infection using RT-qPCR (**Figures 10** **–** **12**). For example, two genes (insulin receptor and aquaporin) were upregulated when *S. schlegelii* was infected with *E. tarda*. CircRNA_4195 showed downregulated expression in response to *E. tarda* infection. Meanwhile, we found that the relative expression of circRNA_2943 was inversely related to the expression from the sequencing result, with upregulated expression in response to *E. tarda* infection at 12 and 24 h. The qRT-PCR results of dre-miR-210-5p showed different expression patterns at the 12 h infection point when compared with that from the Illumina platform. The results showed that the expression trends of most genes in qRT-PCR were in agreement with the sequencing data.

**Figure 11:**
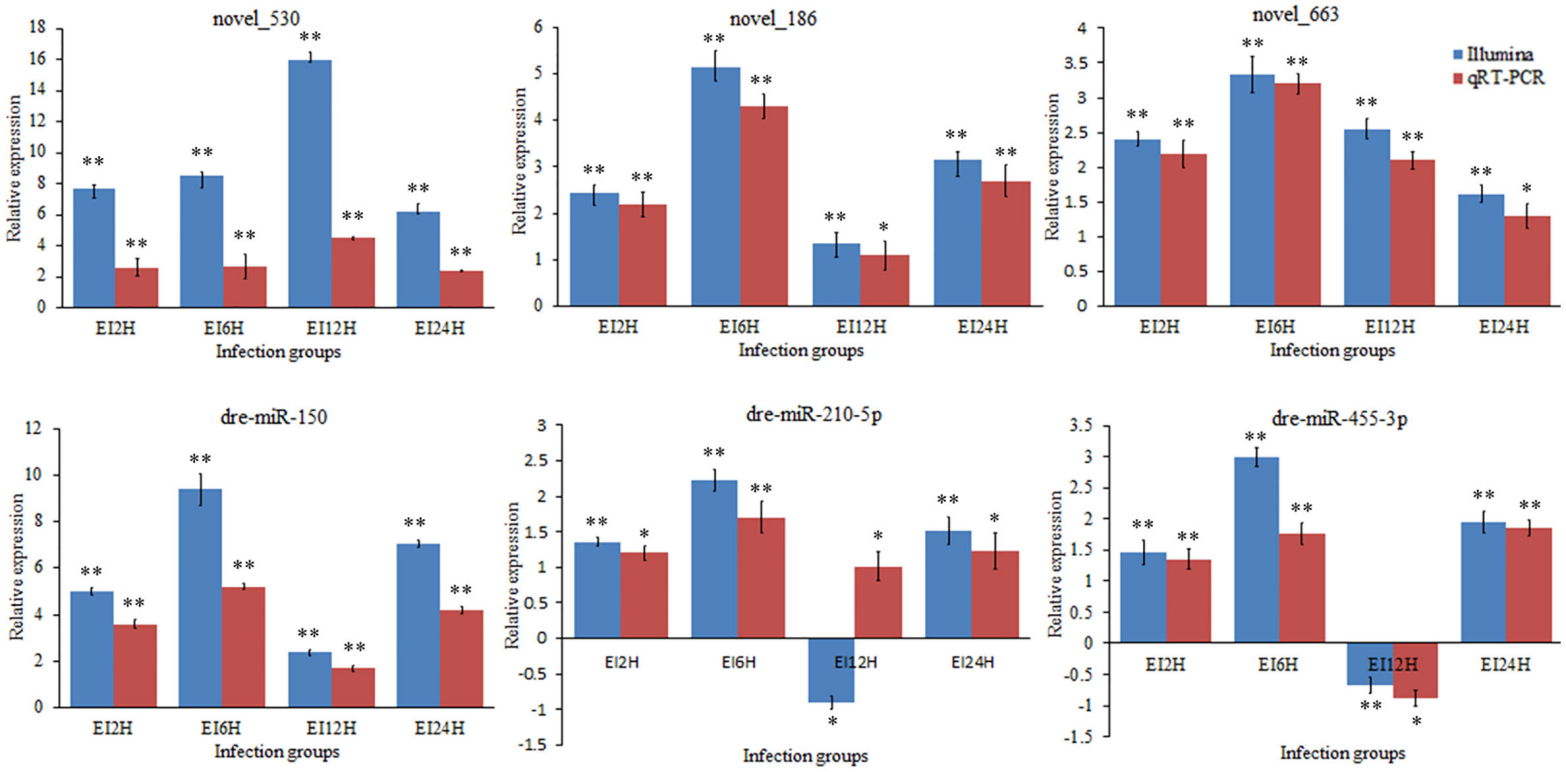
Validation of miRNAs by qRT-PCR analysis. The expression patterns of qRT-PCR were presented between control and, 2, 6, 12, and 24 h infection groups. The results showed the relative fold change and their mean ± standard error (SE) from triplicate experiments. ** indicates significance at the 0.01 level, * indicates significance at the 0.05 level.

**Figure 12:**
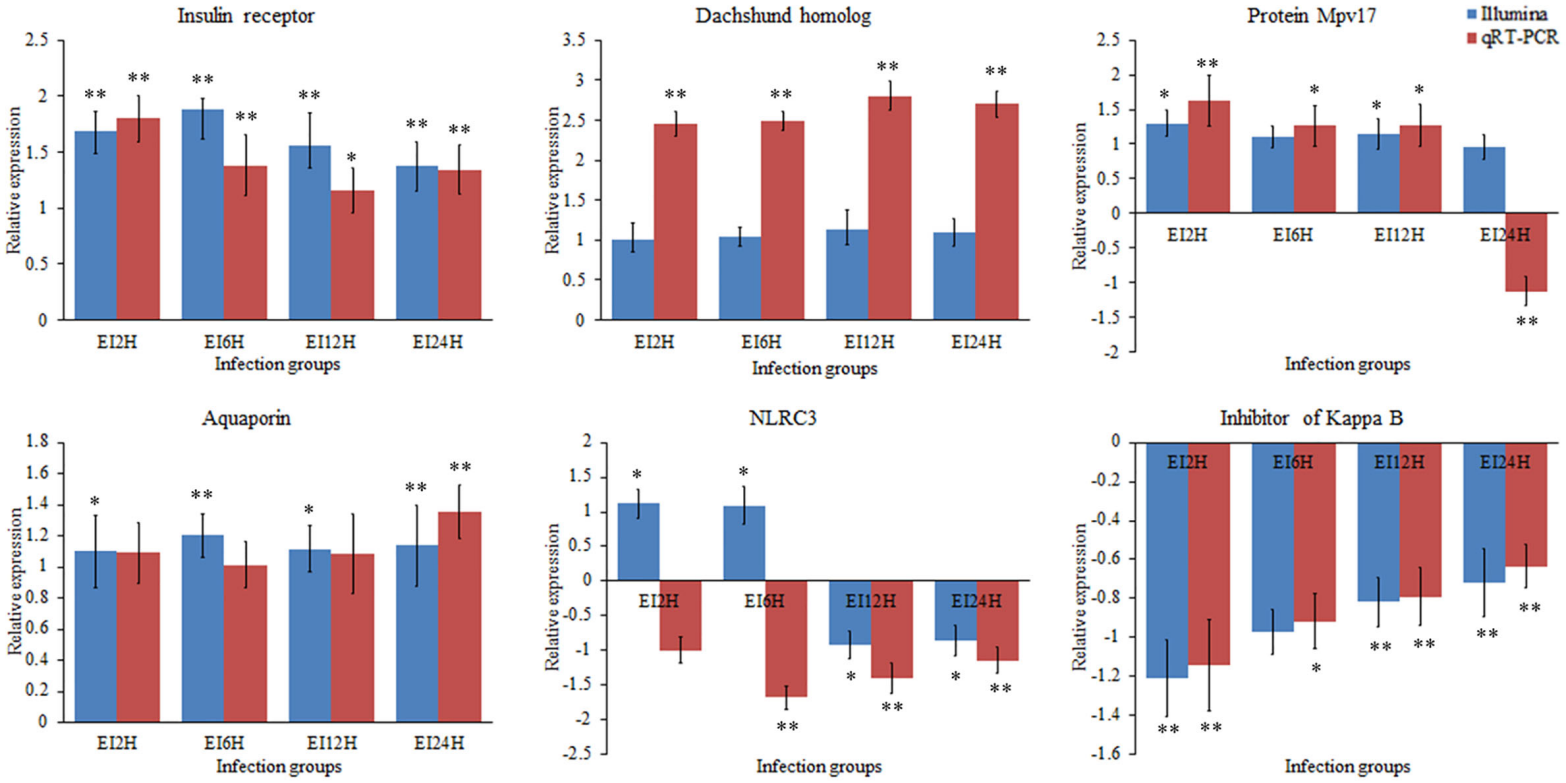
Validation of mRNAs by qRT-PCR analysis. The expression patterns of qRT-PCR were presented between control and, 2, 6, 12, and 24 h infection groups. The results showed the relative fold change and their mean ± standard error (SE) from triplicate experiments. ** indicates significance at the 0.01 level, * indicates significance at the 0.05 level.

## Discussion

The mucosal immune system is the first barrier to defend against the invasion of external pathogens (52). The intestinal mucosa, an important part of the mucosal immune system, plays an important role in the immune response to pathogenic bacteria (53). In our study, *E. tarda* can affect the structure and integrity of the intestinal tissues of *S. schlegelii* in a time-dependent manner. Similar to the histopathological study by Xiu et al. (6), the integrity of the intestinal mucosa structure showed pathological changes such as cell swelling, thickening of the lamina propria, shedding and fragmentation of epithelial cells, and mucosal folds when *P. olivaceus* was infected with *E. tarda*. Furthermore, we found the formation of numerous goblet cells and vacuoles as the infection progressed, which indicated secretion of mucin/mucus, stimulation of immune response, the activity of secondary lysosomes and enzymatic degradation of organelles, and further destruction of the intestine. These histopathological structures demonstrated that *E. tarda* infection changed the physical and integrity barriers of the intestine and stimulated immune responses.

Studies have shown that ncRNAs participate in the interactions between pathogenic microorganisms and teleosts (7, 9, 10, 54, 55). However, studies on the mechanisms of ncRNA regulatory networks in the intestinal mucosal immune response of teleosts are still in infancy. In this study, *S. schlegelii* was used as the research object, *E. tarda* was used as the pathogen to construct an infection model, and the dynamic changes in the transcription level (mRNA) and transcription regulation level (circRNA and miRNA) in the intestine were monitored to explore the circrNA-mirNA-mRNA regulatory network in response to infection. CircRNA is a novel group of ncRNAs with covalently linked closed-loop structures that are generated by reverse splicing events, which are widely expressed in tissues and inhibit the degradation of RNase R enzymes (56). Previous studies have demonstrated that circRNAs can function as miRNA sponges, bind to RNA-binding proteins, and regulate the transcription of target genes and alternative splicing (57). With the development of high-throughput sequencing and bioinformatics techniques, massive amounts of circRNA data from different cells and tissues have been acquired. Most studies on circRNAs are currently about human disease-related genes and in some model species. For example, Memczak et al (2013). proved that the overexpression of ciRS-7/CDR1 could influence the development of the brain by inhibiting the expression of miR-7 (58). Recently, studies have shown that circRNAs are involved in host immune response to pathogenic bacteria in teleost as well as in the developmental process. These studies confirm the existence of circRNA in several species, such as large yellow croaker (*Larimichthys crocea*), *C.semilaevis*, *Oreochromis niloticus*, *Carassius auratus* gibelio, grass carp, and *P. olivaceus* (6, 27, 59–62). However, studies on circRNAs in teleost, especially in intestinal mucosal immune response, are still limited when compared with those in mammals.

In our study, 87 DE-circRNAs were captured at four infection time points in *S. schlegelii* exposed to *E. tarda* infection. Some of these circRNAs were induced once *S. schlegelii* were infected with *E. tarda.* Function analysis showed that the regulated circRNAs were involved in important pathways, such as tight junctions and MAPK signaling pathways, which implied that these circRNAs play vital roles in preventing *S. schlegelii* from *E. tarda* infection. Besides circRNA, miRNAs can regulate the host immune response to various pathogens at the post-transcriptional level by inhibiting mRNA translation or inducing mRNA degradation (13). For example, miRNAs in snout bream, half-smooth tongue sole, and common carp showed different expression patterns when stimulated by pathogenic bacteria or lipopolysaccharides (14, 16, 17). In addition, miRNAs can regulate related signaling pathways by targeting multiple molecules (such as TLR-associated signaling proteins and TLR-induced cytokines) (20). In the present study, we investigated the expression patterns of 79 miRNAs identified at different time points and analyzed their function through their target genes. Analysis of the KEGG metabolic pathway showed that these genes were involved in the metabolic pathway, MAPK signaling pathway, calcium-signaling pathway, protein processing in the endoplasmic reticulum, focal adhesion, tight junction, and apoptosis. These processes also presented a close relationship with immunity. In addition, we found the matching information of mRNA and miRNA, which provided basic data for further research on their regulatory relationships.

In the present study, we systematically analyzed circRNA, miRNA, and mRNA expression profiles in *S. schlegelii* following infection with *E. tarda.* We found that *E. tarda* infection could affect circRNA, miRNA, and mRNA expression profiles. Integrated analyses generated 25 circRNA-miRNA-mRNA interaction networks. Similarly, the potential circRNA-miRNA-mRNA regulatory networks of nectin2, MHC II α-chain, and MHC II β-chains were constructed in the intestine of *P. olivaceus* (6). Fan et al (2019). predicted 2,136 circRNAs in tilapia and analyzed their potential functions by linking them with miRNAs and mRNAs. Meanwhile, we found that the targeted genes in the circRNA-miRNA-mRNA network were involved in immunity-related signaling pathways, such as herpes simplex infection, cell adhesion molecules (CAMs), focal adhesion, tight junction, NOD-like receptor signaling pathway, Toll-like receptor signaling pathway, and NF-κB signaling pathways (61). Interestingly, the novel_circ_0004195/novel_530/*IkB* interaction network was found at all four time points. It has been demonstrated that mammalian nuclear transcription factor NF-κB (nuclear factor of kappa B) family proteins play an important role in the immune system and participate in immune response, tumor formation, and apoptosis by regulating the expression of genes related to lymphocyte development and survival (63). *IκB* is an inhibitor of NF-κB, which keeps NF-κB inactive at rest. Once *IκB* is phosphorylated, it loses its inhibitory effect on NF-κB when cells are triggered by an external signal, and thus can enter the nucleus and regulate gene expression (64). Subsequently, many downstream cytokines and inflammatory factors can be activated to form immune protection for organisms when NF-κB is activated (65). In our study, we found that the expression level of novel_530 increased along the infection time. Thus, it can inhibit the expression of its target gene *IκB*, thereby activating the NF-κB signaling pathway. Therefore, we speculated that novel_circ_0004195/novel_530 might inhibit the expression of *IκB*. Subsequently, the downstream NF-κB signaling pathway was activated. Thus, it can exert the immune barrier function of the intestinal mucosa. In addition, we found networks (novel_circ_0003210/novel_152/apoptosis-stimulating of p53 protein 1 and novel_circ_0001907/novel_127/interleukin-1 receptor type 2) related to apoptosis and interleukin. For this novel_circ_0003210/novel_152/apoptosis-stimulating of p53 protein 1 network, we found that apoptosis-stimulating of p53 protein 1 was upregulated after 6 h of infection. It has been reported that apoptosis-stimulating of p53 protein 1 is an apoptosis-stimulating protein of the p53 (ASPP) family, which contains four ankyrin repeats and an SH3 domain that is involved in protein-protein interactions through the promotion of DNA binding and transactivation of p53-family proteins (66). This implies that the p53 signaling pathway may respond to this infection. Previous studies have reported that the apoptosis and p53 signaling pathways play important roles in immunity (67, 68). Similarly, the induction of interleukin-1 receptor type 2 in the network of novel_circ_0001907/novel_127/interleukin-1 receptor type 2 was observed after infection. We speculate that this network is involved in the immune response in the intestine of *S. schlegelii* because the interleukin 1 receptor family has been demonstrated to play a crucial role in immune responses in the human lung (69). As mentioned in our study, the integrities and morphologies of intestinal tissues of *S. schlegelii* changed with the increasing of infection time. So, what are the associations between histopathological changes and these predicted regulatory networks? Previously, we speculated that novel_530 can relieve the inhibition of *IκB* from the NF-κB signaling pathway by up-regulating its expression. It has been demonstrated that the activation of the NF-κB signaling pathway can active downstream inflammatory factors to cause local inflammation in organism (65). The inflammatory response not only plays a key role in resisting the invasion of pathogens but also induces changes in cell morphologies in organisms (70, 71). Meanwhile, we found that novel_circ_0003210 in the network novel_circ_0003210/novel_152/apoptosis-stimulating of p53 protein 1 is also up-regulated after *E. tarda* infection. Novel_circ_0003210 is a regulator of p53 signaling pathway, which is involved in inducing cell cycle arrest and promoting apoptosis (72). Moreover, numberous reports indicated that the apoptosis present abnormal morphological characteristics, such as karyopyknosis, shrinking cytomembrane, vacuolation, etc (73, 74). Therefore, we speculated that the observation of cell morphologies and even cell death might be related to the regulatory networks of these circRNAs. However, further investigations are still required to determine the targeting relationships of these identified circRNA-miRNA-mRNA networks to understand the immune response and regulatory mechanism of *S. schlegelii*.

## Conclusion

We systematically analyzed circRNAs, miRNAs, and mRNAs in the intestine of *S. schlegelii* after infection with *E. tarda* at different time points (2, 6, 12, and 24 h). Additionally, the corresponding networks of circRNA-miRNA-mRNA were further constructed to investigate their potential roles during *E. tarda* infection. In particular, our results imply that circRNAs play important roles in response to pathogen infection by regulating their related pathways. In total, the integrated analyses generated 25 circRNA-miRNA-mRNA interaction networks, including a novel_circ_0004195/novel_530/*IkB* interaction network, which may exert the immune barrier function by activating the NF-κB signaling pathway in the intestine of *S. schlegelii.* In addition, the circRNA-miRNA-mRNA networks, related to apoptosis (novel_circ_0003210/novel_152/apoptosis-stimulating of p53 protein 1) and interleukin (novel_circ_0001907/novel_127/interleukin-1 receptor type 2), were also identified in our study. Our study indicated that the intestinal immune response of *S. schlegelii* was regulated by circRNAs and miRNAs. However, further studies are needed to explore the mechanism between ncRNAs and mRNAs to better understand the intestinal mucosal immune response in *S. schlegelii*, to better provide theoretical guidance for *S. schlegelii* disease prevention and control.

## Data Availability Statement

Data are available from Dryad at: https://doi.org/10.5061/dryad.7pvmcvdrp.

## Ethics Statement

The animal study was reviewed and approved by Qingdao Agricultural University.

## Author Contributions

MC: analyzed the results and wrote this paper. XY and NY: collected materials and performed the bacteria challenge experiment. QF, TX, and LS: analyzed the sequencing results of circRNA, miRNA, and mRNA. BS: performed the histopathological analysis on the intestine tissues and revised the manuscript. CL and BS: conceived, designed the research, and revised the manuscript. All authors contributed to the article and approved the submitted version.

## Funding

This study was supported by Scientific and Technological Innovation of Blue Granary (2018YFD0900503), Young Experts of Taishan Scholars (NO.tsqn201909130), Science and Technology Support Plan for Youth Innovation of Colleges and Universities in Shandong Province (2019KJF003), the “First Class Fishery Discipline” Programme in Shandong Province, a special talent programme “One Thing One Decision (Yishi Yiyi)” Programme in Shandong Province, China, Breeding Plan of Shandong Provincial Qingchuang Research Team (2019), and Breeding Plan of Shandong Provincial Qingchuang Research Team (2019).

## Conflict of Interest

The authors declare that the research was conducted in the absence of any commercial or financial relationships that could be construed as a potential conflict of interest.

## Supplementary Material

Supplementary Figure 1 Venn diagram of core GO terms at four post-infections.

Supplementary Figure 2 Mapping reads of small RNAs on the *S. schlegelii* genome.
