# Supplementary figures and images for: Integrated Analysis of circRNA-miRNA-mRNA Regulatory Networks in the Intestine of Sebastes schlegelii Following Edwardsiella tarda Challenge
Source: Front Immunol. 2021 Jan 20;11:618687. doi: 10.3389/fimmu.2020.618687 (PMC7857051; doi:10.3389/fimmu.2020.618687)

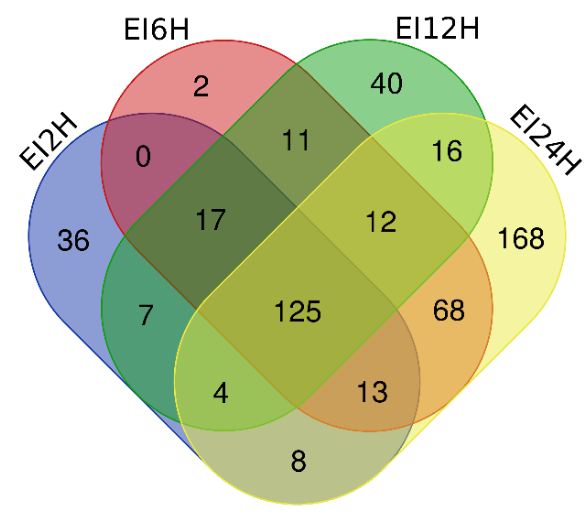

Supplement: Supplementary Figure 1 — Venn diagram of core GO terms at four post-infections. [file Image_1.png]

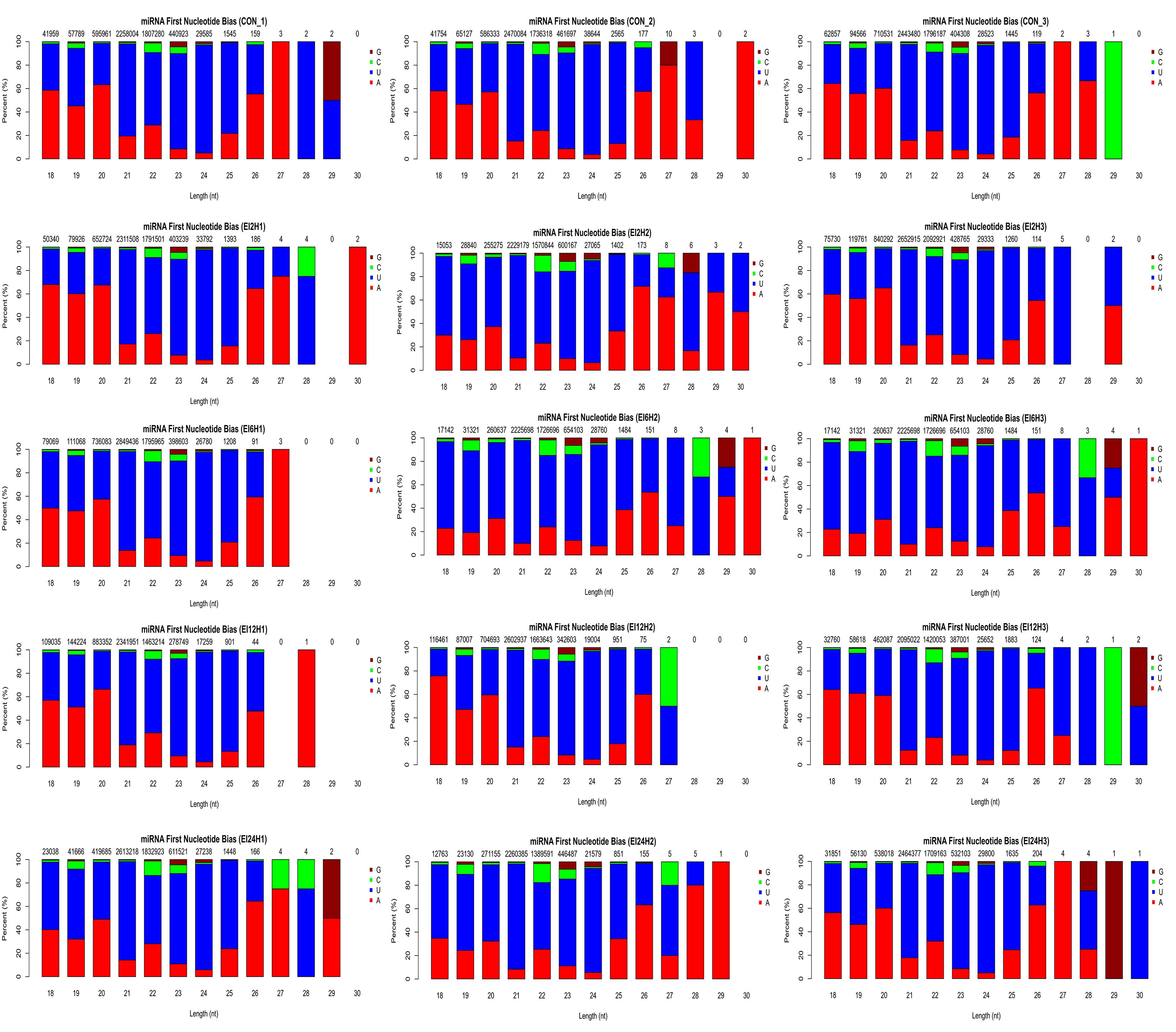

Supplement: Supplementary Figure 2 — Mapping reads of small RNAs on the S. schlegelii genome. [file Image_2.tif]
