# Supplementary material for: Integrated Analysis of circRNA-miRNA-mRNA Regulatory Networks in the Intestine of Sebastes schlegelii Following Edwardsiella tarda Challenge
Source: Front Immunol. 2021 Jan 20;11:618687. doi: 10.3389/fimmu.2020.618687 (PMC7857051; doi:10.3389/fimmu.2020.618687)
Supplement: Supplementary file 5 [file Table_1.docx]

Table S1 Primers used in this study

| Gene name | Forward primers | Reverse primers |
| --- | --- | --- |
| Insulin receptor | AACCCGGATCTCTGCTATCT | CCACACTCTCTGTCGTTCTTG |
| Dachshund homolog | CCTCAACACCATCGCTAACA | CACACACTCTCTCCTTGATGAC |
| Protein Mpv17 | CACAATCCTGCACTTAGCAATC | GCAACGGGCTCTGGTATAAA |
| Aquaporin | GACCCTGCTCACCAACATAC | CATCTTCAGCTCTCGCTTCTC |
| NLRC3 | AGGAGAGTCCGTGATGAAGA | CAGCAGAAGACTGGGATTAGAC |
| Inhibitor of κ B | CTACGGGCCTGAAGCAAATA | GGAGAAGTTGTAGTCTGCCATAA |
| circRNA_729 | GCAAACAGAGTCGAGGAAGAG | TGACACTCGTGCTTCTTAACC |
| circRNA_2647 | GGCCTCAGCGTGGATAG | GTGGACAGAAGGTTGTGGA |
| circRNA_2943 | CTGGCTAACAGCAGGAACAA | CGTGGCTTAGGAAAGGAAACA |
| circRNA_3141 | GACTCTTCACCAAAGCCAGA | GCCCAGTATGACGGATAGATAAG |
| circRNA_3199 | TCCTCCTGGATACTACGTCTTC | CGGTCCTGGTTCAACTTCTT |
| circRNA_4195 | CTCCATCATGCCCATCTTCAA | CGTTGATGAGCGACTCCTTAC |
| novel_530 | TCGGACCGCTGTTGGAGACGA |  |
| novel_186 | CATCGCGAGGGTGAAAGTGTGT |  |
| novel_663 | TCACGGTCGGAGACGATAAAGT |  |
| dre-miR-150 | TCTCCCAATCCTTGTACCAGTG |  |
| dre-miR-210-5p | AGCCACTGACTAACGCACATTG |  |
| dre-miR-455-3p | ATGCAGTCCATGGGCATATACAC |  |
| RPL17 | AGGCGACGCACCTACCG | TCTGGTTTGGGGACGA |
