# Supplementary material for: Integrated Analysis of circRNA-miRNA-mRNA Regulatory Networks in the Intestine of Sebastes schlegelii Following Edwardsiella tarda Challenge
Source: Front Immunol. 2021 Jan 20;11:618687. doi: 10.3389/fimmu.2020.618687 (PMC7857051; doi:10.3389/fimmu.2020.618687)
Supplement: Supplementary file 6 [file Table_2.docx]

| Sample  name | Raw  reads | Clean  reads | Raw  bases(G) | Clean  bases(G) | Error  rate (%) | Q20  (%) | Q30  (%) | GC  Content (%) |
| --- | --- | --- | --- | --- | --- | --- | --- | --- |
| CON1 | 109,209,574 | 106,999,156 | 16.38 | 16.05 | 0.02 | 98.02 | 94.19 | 52.41 |
| CON2 | 131,090,972 | 128,099,084 | 19.66 | 19.21 | 0.02 | 98.04 | 94.24 | 53.78 |
| CON3 | 249,862,106 | 242,141,850 | 37.48 | 36.32 | 0.03 | 97.98 | 94.16 | 56.44 |
| EI2H1 | 187,375,326 | 182,946,264 | 28.11 | 27.44 | 0.03 | 97.96 | 94.12 | 55.65 |
| EI2H2 | 171,895,520 | 169,804,116 | 25.78 | 25.47 | 0.03 | 98.02 | 94.2 | 54.74 |
| EI2H3 | 219,322,248 | 215,487,754 | 32.9 | 32.32 | 0.02 | 98.07 | 94.3 | 55.89 |
| EI6H1 | 119,343,946 | 117,508,372 | 17.9 | 17.63 | 0.02 | 98.00 | 94.23 | 52.78 |
| EI6H2 | 178,037,386 | 171,143,610 | 26.71 | 25.67 | 0.02 | 98.22 | 94.78 | 54.02 |
| EI6H3 | 219,751,300 | 214,816,378 | 32.96 | 32.22 | 0.02 | 98.22 | 94.75 | 54.47 |
| EI12H1 | 101,466,464 | 100,415,642 | 15.22 | 15.06 | 0.03 | 98.00 | 94.17 | 52.41 |
| EI12H2 | 128,797,640 | 127,017,956 | 19.32 | 19.05 | 0.03 | 97.93 | 94.07 | 54.49 |
| EI12H3 | 197,468,382 | 193,783,576 | 29.62 | 29.07 | 0.02 | 98.25 | 94.84 | 55.37 |
| EI24H1 | 108,532,820 | 107,178,698 | 16.28 | 16.08 | 0.03 | 97.99 | 94.15 | 52.18 |
| EI24H2 | 190,163,690 | 187,043,368 | 28.52 | 28.06 | 0.02 | 98.18 | 94.65 | 54.68 |
| EI24H3 | 133,224,358 | 130,620,804 | 19.98 | 19.59 | 0.02 | 98.12 | 94.51 | 53.24 |

**Table S2 Overview of circRNA sequencing data**
