# Supplementary material for: Integrated Analysis of circRNA-miRNA-mRNA Regulatory Networks in the Intestine of Sebastes schlegelii Following Edwardsiella tarda Challenge
Source: Front Immunol. 2021 Jan 20;11:618687. doi: 10.3389/fimmu.2020.618687 (PMC7857051; doi:10.3389/fimmu.2020.618687)
Supplement: Supplementary file 7 [file Table_3.docx]

**Table S3 Overview of miRNA sequencing data**

| Sample | Reads | Bases | Error rate | Q20 | Q30 | GC content |
| --- | --- | --- | --- | --- | --- | --- |
| CON1 | 11499017 | 0.575G | 0.01% | 98.22% | 95.67% | 49.09% |
| CON2 | 12514478 | 0.626G | 0.01% | 98.23% | 95.70% | 49.07% |
| CON3 | 11506288 | 0.575G | 0.01% | 98.16% | 95.54% | 48.86% |
| EI2H1 | 11382881 | 0.569G | 0.01% | 98.19% | 95.60% | 48.90% |
| EI2H2 | 10956647 | 0.548G | 0.01% | 98.08% | 95.31% | 49.79% |
| EI2H3 | 12722619 | 0.636G | 0.01% | 97.98% | 94.99% | 48.84% |
| EI6H1 | 12746970 | 0.637G | 0.01% | 97.97% | 95.01% | 49.02% |
| EI6H2 | 11732354 | 0.587G | 0.01% | 98.08% | 95.30% | 49.72% |
| EI6H3 | 11732354 | 0.587G | 0.01% | 98.08% | 95.30% | 49.72% |
| EI12H1 | 11981165 | 0.599G | 0.01% | 97.88% | 94.75% | 48.62% |
| EI12H2 | 10331966 | 0.517G | 0.01% | 98.07% | 95.23% | 49.05% |
| EI12H3 | 11212627 | 0.561G | 0.01% | 97.89% | 94.83% | 49.38% |
| EI24H1 | 11474270 | 0.574G | 0.01% | 98.02% | 95.12% | 49.47% |
| EI24H2 | 10226678 | 0.511G | 0.01% | 98.14% | 95.49% | 49.71% |
| EI24H3 | 10500100 | 0.525G | 0.01% | 98.09% | 95.29% | 49.28% |
