# Supplementary material for: Integrated Analysis of circRNA-miRNA-mRNA Regulatory Networks in the Intestine of Sebastes schlegelii Following Edwardsiella tarda Challenge
Source: Front Immunol. 2021 Jan 20;11:618687. doi: 10.3389/fimmu.2020.618687 (PMC7857051; doi:10.3389/fimmu.2020.618687)
Supplement: Supplementary file 8 [file Table_4.docx]

Table S4 Statistics of small RNA reads mapped to genome

| Sample | Total sRNA | Mapped sRNA(+) | Mapped sRNA(-) | Mapped sRNA |
| --- | --- | --- | --- | --- |
| CON1 | 10952241 (100.00%) | 10240755 (93.50%) | 2767309 (25.27%) | 7473446 (68.24%) |
| CON2 | 11871197 (100.00%) | 11103729 (93.54%) | 3055092 (25.74%) | 8048637 (67.80%) |
| CON3 | 10854555 (100.00%) | 10229429 (94.24%) | 2597776 (23.93%) | 7631653 (70.31%) |
| EI2H1 | 10831505 (100.00%) | 10162106 (93.82%) | 2644520 (24.42%) | 7517586 (69.40%) |
| EI2H2 | 10455131 (100.00%) | 9690053 (92.68%) | 3302518 (31.59%) | 6387535 (61.09%) |
| EI2H3 | 11934189 (100.00%) | 11163501 (93.54%) | 2963559 (24.83%) | 8199942 (68.71%) |
| EI6H1 | 11760851 (100.00%) | 11002550 (93.55%) | 3023065 (25.70%) | 7979485 (67.85%) |
| EI6H2 | 11277649 (100.00%) | 10401196 (92.23%) | 3424334 (30.36%) | 6976862 (61.86%) |
| EI6H3 | 11277649 (100.00%) | 10401196 (92.23%) | 3424334 (30.36%) | 6976862 (61.86%) |
| EI12H1 | 10524348 (100.00%) | 9807211 (93.19%) | 2098740 (19.94%) | 7708471 (73.24%) |
| EI12H2 | 9584485 (100.00%) | 9084797 (94.79%) | 2383198 (24.87%) | 6701599 (69.92%) |
| EI12H3 | 10462476 (100.00%) | 9630286 (92.05%) | 3455867 (33.03%) | 6174419 (59.01%) |
| EI24H1 | 11001893 (100.00%) | 10316782 (93.77%) | 3081098 (28.01%) | 7235684 (65.77%) |
| EI24H2 | 9808329 (100.00%) | 9151144 (93.30%) | 2444797 (24.93%) | 6706347 (68.37%) |
| EI24H3 | 10031877 (100.00%) | 9445442 (94.15%) | 2618503 (26.10%) | 6826939 (68.05%) |
