# Supplementary material for: Integrated Analysis of circRNA-miRNA-mRNA Regulatory Networks in the Intestine of Sebastes schlegelii Following Edwardsiella tarda Challenge
Source: Front Immunol. 2021 Jan 20;11:618687. doi: 10.3389/fimmu.2020.618687 (PMC7857051; doi:10.3389/fimmu.2020.618687)
Supplement: Supplementary file 9 [file Table_5.docx]

Table S5 miRNA and circRNA pairs

| miRNA | circRNA |  |
| --- | --- | --- |
| ccr-miR-101b | novel_circ_0001954 | |
| ccr-miR-101b | novel_circ_0001528 | |
| ccr-miR-10b | novel_circ_0003247 | |
| ccr-miR-10b | novel_circ_0000789 | |
| ccr-miR-10b | novel_circ_0000246 | |
| ccr-miR-10b | novel_circ_0000250 | |
| ccr-miR-10d | novel_circ_0003247 | |
| ccr-miR-10d | novel_circ_0000789 | |
| ccr-miR-10d | novel_circ_0000246 | |
| ccr-miR-10d | novel_circ_0000250 | |
| ccr-miR-122 | novel_circ_0002453 | |
| ccr-miR-122 | novel_circ_0002364 | |
| ccr-miR-122 | novel_circ_0003879 | |
| ccr-miR-122 | novel_circ_0002922 | |
| ccr-miR-122 | novel_circ_0004124 | |
| ccr-miR-122 | novel_circ_0001528 | |
| ccr-miR-128 | novel_circ_0002653 | |
| ccr-miR-128 | novel_circ_0003210 | |
| ccr-miR-128 | novel_circ_0002944 | |
| ccr-miR-128 | novel_circ_0001954 | |
| ccr-miR-128 | novel_circ_0002031 | |
| ccr-miR-128 | novel_circ_0000577 | |
| ccr-miR-128 | novel_circ_0000180 | |
| ccr-miR-129 | novel_circ_0002490 | |
| ccr-miR-129 | novel_circ_0003372 | |
| ccr-miR-129 | novel_circ_0003246 | |
| ccr-miR-129 | novel_circ_0002842 | |
| ccr-miR-129 | novel_circ_0001544 | |
| ccr-miR-133a-3p | novel_circ_0002537 | |
| ccr-miR-133a-3p | novel_circ_0003853 | |
| ccr-miR-133a-3p | novel_circ_0002922 | |
| ccr-miR-133a-3p | novel_circ_0004002 | |
| ccr-miR-133a-3p | novel_circ_0000636 | |
| ccr-miR-133a-3p | novel_circ_0000484 | |
| ccr-miR-133a-3p | novel_circ_0000340 | |
| ccr-miR-133a-3p | novel_circ_0001370 | |
| ccr-miR-139 | novel_circ_0003775 | |
| ccr-miR-139 | novel_circ_0003560 | |
| ccr-miR-139 | novel_circ_0002774 | |
| ccr-miR-139 | novel_circ_0002774 | |
| ccr-miR-16c | novel_circ_0002506 | |
| ccr-miR-16c | novel_circ_0003775 | |
| ccr-miR-16c | novel_circ_0002744 | |
| ccr-miR-16c | novel_circ_0002865 | |
| ccr-miR-16c | novel_circ_0002869 | |
| ccr-miR-16c | novel_circ_0000741 | |
| ccr-miR-16c | novel_circ_0001200 | |
| ccr-miR-16c | novel_circ_0001019 | |
| ccr-miR-16c | novel_circ_0001041 | |
| ccr-miR-16c | novel_circ_0001659 | |
| ccr-miR-16c | novel_circ_0001544 | |
| ccr-miR-181a | novel_circ_0002117 | |
| ccr-miR-181a | novel_circ_0003210 | |
| ccr-miR-181a | novel_circ_0000731 | |
| ccr-miR-181a | novel_circ_0000250 | |
| ccr-miR-190 | novel_circ_0002732 | |
| ccr-miR-218b | novel_circ_0003828 | |
| ccr-miR-218b | novel_circ_0003583 | |
| ccr-miR-218b | novel_circ_0003246 | |
| ccr-miR-218b | novel_circ_0003247 | |
| ccr-miR-218b | novel_circ_0001615 | |
| ccr-miR-23a | novel_circ_0002482 | |
| ccr-miR-23a | novel_circ_0002117 | |
| ccr-miR-23a | novel_circ_0003609 | |
| ccr-miR-23a | novel_circ_0003189 | |
| ccr-miR-23a | novel_circ_0001780 | |
| ccr-miR-23a | novel_circ_0001954 | |
| ccr-miR-23a | novel_circ_0000651 | |
| ccr-miR-23b | novel_circ_0002482 | |
| ccr-miR-23b | novel_circ_0002117 | |
| ccr-miR-23b | novel_circ_0003609 | |
| ccr-miR-23b | novel_circ_0003189 | |
| ccr-miR-23b | novel_circ_0001780 | |
| ccr-miR-23b | novel_circ_0001954 | |
| ccr-miR-23b | novel_circ_0000651 | |
| ccr-miR-489 | novel_circ_0003865 | |
| ccr-miR-489 | novel_circ_0003879 | |
| ccr-miR-489 | novel_circ_0000192 | |
| ccr-miR-489 | novel_circ_0001041 | |
| ccr-miR-722 | novel_circ_0003372 | |
| ccr-miR-722 | novel_circ_0002842 | |
| ccr-miR-9-3p | novel_circ_0002419 | |
| ccr-miR-9-3p | novel_circ_0002516 | |
| ccr-miR-9-3p | novel_circ_0002534 | |
| ccr-miR-9-3p | novel_circ_0002117 | |
| ccr-miR-9-3p | novel_circ_0003842 | |
| ccr-miR-9-3p | novel_circ_0003247 | |
| ccr-miR-9-3p | novel_circ_0002999 | |
| ccr-miR-9-3p | novel_circ_0002869 | |
| ccr-miR-9-3p | novel_circ_0002031 | |
| ccr-miR-9-3p | novel_circ_0000636 | |
| ccr-miR-9-3p | novel_circ_0000182 | |
| ccr-miR-9-3p | novel_circ_0001544 | |
| dre-let-7b | novel_circ_0004265 | |
| dre-let-7b | novel_circ_0003599 | |
| dre-let-7b | novel_circ_0002744 | |
| dre-let-7b | novel_circ_0001907 | |
| dre-let-7b | novel_circ_0000789 | |
| dre-let-7b | novel_circ_0001544 | |
| dre-let-7c-5p | novel_circ_0004265 | |
| dre-let-7c-5p | novel_circ_0003599 | |
| dre-let-7c-5p | novel_circ_0002744 | |
| dre-let-7c-5p | novel_circ_0001907 | |
| dre-let-7c-5p | novel_circ_0000789 | |
| dre-let-7c-5p | novel_circ_0001544 | |
| dre-let-7d-5p | novel_circ_0004265 | |
| dre-let-7d-5p | novel_circ_0003599 | |
| dre-let-7d-5p | novel_circ_0002744 | |
| dre-let-7d-5p | novel_circ_0001907 | |
| dre-let-7d-5p | novel_circ_0000789 | |
| dre-let-7d-5p | novel_circ_0001544 | |
| dre-miR-1 | novel_circ_0002490 | |
| dre-miR-101a | novel_circ_0001022 | |
| dre-miR-101b | novel_circ_0001022 | |
| dre-miR-10a-5p | novel_circ_0003247 | |
| dre-miR-10a-5p | novel_circ_0000789 | |
| dre-miR-10a-5p | novel_circ_0000246 | |
| dre-miR-10a-5p | novel_circ_0000250 | |
| dre-miR-10b-5p | novel_circ_0003247 | |
| dre-miR-10b-5p | novel_circ_0000789 | |
| dre-miR-10b-5p | novel_circ_0000246 | |
| dre-miR-10b-5p | novel_circ_0000250 | |
| dre-miR-10b-2-3p | novel_circ_0001659 | |
| dre-miR-10c-5p | novel_circ_0003247 | |
| dre-miR-10c-5p | novel_circ_0000789 | |
| dre-miR-10c-5p | novel_circ_0000246 | |
| dre-miR-10c-5p | novel_circ_0000250 | |
| dre-miR-10d-5p | novel_circ_0003247 | |
| dre-miR-10d-5p | novel_circ_0000789 | |
| dre-miR-10d-5p | novel_circ_0000246 | |
| dre-miR-10d-5p | novel_circ_0000250 | |
| dre-miR-122 | novel_circ_0002453 | |
| dre-miR-122 | novel_circ_0002364 | |
| dre-miR-122 | novel_circ_0003879 | |
| dre-miR-122 | novel_circ_0002922 | |
| dre-miR-122 | novel_circ_0004124 | |
| dre-miR-122 | novel_circ_0001528 | |
| dre-miR-125a | novel_circ_0001094 | |
| dre-miR-125b-5p | novel_circ_0001094 | |
| dre-miR-125c-5p | novel_circ_0001094 | |
| dre-miR-128-3p | novel_circ_0002653 | |
| dre-miR-128-3p | novel_circ_0003210 | |
| dre-miR-128-3p | novel_circ_0002944 | |
| dre-miR-128-3p | novel_circ_0001954 | |
| dre-miR-128-3p | novel_circ_0002031 | |
| dre-miR-128-3p | novel_circ_0000577 | |
| dre-miR-128-3p | novel_circ_0000180 | |
| dre-miR-129-5p | novel_circ_0002490 | |
| dre-miR-129-5p | novel_circ_0003372 | |
| dre-miR-129-5p | novel_circ_0003246 | |
| dre-miR-129-5p | novel_circ_0002842 | |
| dre-miR-129-5p | novel_circ_0001544 | |
| dre-miR-129-1-3p | novel_circ_0002514 | |
| dre-miR-129-1-3p | novel_circ_0002338 | |
| dre-miR-129-1-3p | novel_circ_0003879 | |
| dre-miR-129-1-3p | novel_circ_0002869 | |
| dre-miR-129-1-3p | novel_circ_0000262 | |
| dre-miR-129-1-3p | novel_circ_0000192 | |
| dre-miR-129-3-3p | novel_circ_0000484 | |
| dre-miR-129-3-3p | novel_circ_0000262 | |
| dre-miR-129-3-3p | novel_circ_0001238 | |
| dre-miR-129-3p | novel_circ_0000484 | |
| dre-miR-129-3p | novel_circ_0000262 | |
| dre-miR-129-3p | novel_circ_0001238 | |
| dre-miR-133a-3p | novel_circ_0002516 | |
| dre-miR-133a-3p | novel_circ_0004002 | |
| dre-miR-133a-3p | novel_circ_0000180 | |
| dre-miR-133a-3p | novel_circ_0001370 | |
| dre-miR-135a | novel_circ_0000577 | |
| dre-miR-135a | novel_circ_0001391 | |
| dre-miR-135c | novel_circ_0000577 | |
| dre-miR-135c | novel_circ_0001391 | |
| dre-miR-1388-3p | novel_circ_0004265 | |
| dre-miR-1388-3p | novel_circ_0002338 | |
| dre-miR-1388-3p | novel_circ_0003853 | |
| dre-miR-1388-3p | novel_circ_0003960 | |
| dre-miR-1388-3p | novel_circ_0003966 | |
| dre-miR-1388-3p | novel_circ_0003055 | |
| dre-miR-1388-3p | novel_circ_0004136 | |
| dre-miR-139-5p | novel_circ_0003775 | |
| dre-miR-139-5p | novel_circ_0003560 | |
| dre-miR-139-5p | novel_circ_0002774 | |
| dre-miR-139-5p | novel_circ_0002774 | |
| dre-miR-142a-3p | novel_circ_0001041 | |
| dre-miR-144-3p | novel_circ_0001954 | |
| dre-miR-145-5p | novel_circ_0002534 | |
| dre-miR-145-5p | novel_circ_0002398 | |
| dre-miR-145-5p | novel_circ_0003828 | |
| dre-miR-145-5p | novel_circ_0003879 | |
| dre-miR-145-5p | novel_circ_0003372 | |
| dre-miR-145-5p | novel_circ_0003189 | |
| dre-miR-145-5p | novel_circ_0003245 | |
| dre-miR-145-5p | novel_circ_0001907 | |
| dre-miR-145-5p | novel_circ_0000577 | |
| dre-miR-145-5p | novel_circ_0000250 | |
| dre-miR-145-5p | novel_circ_0001241 | |
| dre-miR-145-5p | novel_circ_0001054 | |
| dre-miR-145-5p | novel_circ_0000971 | |
| dre-miR-148 | novel_circ_0004265 | |
| dre-miR-148 | novel_circ_0003828 | |
| dre-miR-148 | novel_circ_0003210 | |
| dre-miR-150 | novel_circ_0003372 | |
| dre-miR-150 | novel_circ_0003210 | |
| dre-miR-152 | novel_circ_0004265 | |
| dre-miR-152 | novel_circ_0003828 | |
| dre-miR-152 | novel_circ_0003210 | |
| dre-miR-153a-3p | novel_circ_0001907 | |
| dre-miR-153c-3p | novel_circ_0001907 | |
| dre-miR-16c-5p | novel_circ_0002506 | |
| dre-miR-16c-5p | novel_circ_0003775 | |
| dre-miR-16c-5p | novel_circ_0002744 | |
| dre-miR-16c-5p | novel_circ_0002865 | |
| dre-miR-16c-5p | novel_circ_0002869 | |
| dre-miR-16c-5p | novel_circ_0000741 | |
| dre-miR-16c-5p | novel_circ_0001200 | |
| dre-miR-16c-5p | novel_circ_0001019 | |
| dre-miR-16c-5p | novel_circ_0001041 | |
| dre-miR-16c-5p | novel_circ_0001659 | |
| dre-miR-16c-5p | novel_circ_0001544 | |
| dre-miR-1788-5p | novel_circ_0003891 | |
| dre-miR-1788-5p | novel_circ_0004002 | |
| dre-miR-1788-5p | novel_circ_0000259 | |
| dre-miR-1788-5p | novel_circ_0001054 | |
| dre-miR-1788-3p | novel_circ_0002331 | |
| dre-miR-1788-3p | novel_circ_0002744 | |
| dre-miR-1788-3p | novel_circ_0002865 | |
| dre-miR-1788-3p | novel_circ_0001958 | |
| dre-miR-1788-3p | novel_circ_0000180 | |
| dre-miR-1788-3p | novel_circ_0001258 | |
| dre-miR-1788-3p | novel_circ_0001528 | |
| dre-miR-181a-5p | novel_circ_0002117 | |
| dre-miR-181a-5p | novel_circ_0003210 | |
| dre-miR-181a-5p | novel_circ_0000731 | |
| dre-miR-181a-5p | novel_circ_0000250 | |
| dre-miR-181a-3p | novel_circ_0000724 | |
| dre-miR-181a-3p | novel_circ_0001283 | |
| dre-miR-181a-5-3p | novel_circ_0000724 | |
| dre-miR-181a-5-3p | novel_circ_0001283 | |
| dre-miR-184 | novel_circ_0003853 | |
| dre-miR-184 | novel_circ_0003599 | |
| dre-miR-184 | novel_circ_0001744 | |
| dre-miR-18a | novel_circ_0002695 | |
| dre-miR-18a | novel_circ_0002696 | |
| dre-miR-18a | novel_circ_0003229 | |
| dre-miR-18a | novel_circ_0004136 | |
| dre-miR-18a | novel_circ_0000731 | |
| dre-miR-18a | novel_circ_0001022 | |
| dre-miR-18a | novel_circ_0001528 | |
| dre-miR-190a | novel_circ_0002732 | |
| dre-miR-190b | novel_circ_0002732 | |
| dre-miR-196a-5p | novel_circ_0004265 | |
| dre-miR-196a-5p | novel_circ_0003966 | |
| dre-miR-196a-5p | novel_circ_0002744 | |
| dre-miR-196a-5p | novel_circ_0000789 | |
| dre-miR-196b | novel_circ_0004265 | |
| dre-miR-196b | novel_circ_0003966 | |
| dre-miR-196b | novel_circ_0002744 | |
| dre-miR-196b | novel_circ_0000789 | |
| dre-miR-199-5p | novel_circ_0002534 | |
| dre-miR-199-5p | novel_circ_0002537 | |
| dre-miR-199-5p | novel_circ_0002244 | |
| dre-miR-199-5p | novel_circ_0003234 | |
| dre-miR-199-5p | novel_circ_0000473 | |
| dre-miR-199-5p | novel_circ_0000474 | |
| dre-miR-199-5p | novel_circ_0000482 | |
| dre-miR-19a-3p | novel_circ_0004267 | |
| dre-miR-19a-3p | novel_circ_0002514 | |
| dre-miR-19b-5p | novel_circ_0003966 | |
| dre-miR-19b-5p | novel_circ_0000737 | |
| dre-miR-19b-5p | novel_circ_0000482 | |
| dre-miR-19b-3p | novel_circ_0004267 | |
| dre-miR-19b-3p | novel_circ_0002514 | |
| dre-miR-19c-3p | novel_circ_0004267 | |
| dre-miR-19c-3p | novel_circ_0002514 | |
| dre-miR-19d-3p | novel_circ_0004267 | |
| dre-miR-19d-3p | novel_circ_0002514 | |
| dre-miR-203a-3p | novel_circ_0001819 | |
| dre-miR-205-5p | novel_circ_0002534 | |
| dre-miR-205-5p | novel_circ_0002203 | |
| dre-miR-205-5p | novel_circ_0002244 | |
| dre-miR-205-5p | novel_circ_0002255 | |
| dre-miR-205-5p | novel_circ_0003372 | |
| dre-miR-205-5p | novel_circ_0002842 | |
| dre-miR-205-5p | novel_circ_0001842 | |
| dre-miR-205-5p | novel_circ_0000259 | |
| dre-miR-205-5p | novel_circ_0001258 | |
| dre-miR-206-3p | novel_circ_0002490 | |
| dre-miR-20a-5p | novel_circ_0002455 | |
| dre-miR-20a-5p | novel_circ_0000473 | |
| dre-miR-21 | novel_circ_0002490 | |
| dre-miR-210-5p | novel_circ_0002255 | |
| dre-miR-210-5p | novel_circ_0003189 | |
| dre-miR-210-5p | novel_circ_0003142 | |
| dre-miR-210-5p | novel_circ_0002766 | |
| dre-miR-210-5p | novel_circ_0001784 | |
| dre-miR-210-5p | novel_circ_0001959 | |
| dre-miR-210-5p | novel_circ_0001041 | |
| dre-miR-212-5p | novel_circ_0002695 | |
| dre-miR-212-5p | novel_circ_0002696 | |
| dre-miR-212-5p | novel_circ_0002117 | |
| dre-miR-212-5p | novel_circ_0002395 | |
| dre-miR-212-5p | novel_circ_0003221 | |
| dre-miR-212-5p | novel_circ_0003254 | |
| dre-miR-212-5p | novel_circ_0000778 | |
| dre-miR-217 | novel_circ_0002482 | |
| dre-miR-217 | novel_circ_0003744 | |
| dre-miR-2184 | novel_circ_0001528 | |
| dre-miR-218a | novel_circ_0003828 | |
| dre-miR-218a | novel_circ_0003583 | |
| dre-miR-218a | novel_circ_0003246 | |
| dre-miR-218a | novel_circ_0003247 | |
| dre-miR-218a | novel_circ_0001615 | |
| dre-miR-218b | novel_circ_0003828 | |
| dre-miR-218b | novel_circ_0003583 | |
| dre-miR-218b | novel_circ_0003246 | |
| dre-miR-218b | novel_circ_0003247 | |
| dre-miR-218b | novel_circ_0001615 | |
| dre-miR-221-5p | novel_circ_0002730 | |
| dre-miR-221-5p | novel_circ_0002453 | |
| dre-miR-221-5p | novel_circ_0003828 | |
| dre-miR-221-5p | novel_circ_0003254 | |
| dre-miR-221-5p | novel_circ_0003344 | |
| dre-miR-221-5p | novel_circ_0002744 | |
| dre-miR-221-5p | novel_circ_0001784 | |
| dre-miR-221-5p | novel_circ_0000789 | |
| dre-miR-221-5p | novel_circ_0000192 | |
| dre-miR-221-5p | novel_circ_0001258 | |
| dre-miR-223 | novel_circ_0002740 | |
| dre-miR-22b-5p | novel_circ_0002096 | |
| dre-miR-22b-5p | novel_circ_0002189 | |
| dre-miR-22b-5p | novel_circ_0002203 | |
| dre-miR-22b-5p | novel_circ_0002398 | |
| dre-miR-22b-5p | novel_circ_0001954 | |
| dre-miR-22b-5p | novel_circ_0004002 | |
| dre-miR-22b-5p | novel_circ_0000577 | |
| dre-miR-22b-5p | novel_circ_0000192 | |
| dre-miR-22b-5p | novel_circ_0001094 | |
| dre-miR-22b-5p | novel_circ_0000837 | |
| dre-miR-23a-3p | novel_circ_0002482 | |
| dre-miR-23a-3p | novel_circ_0002117 | |
| dre-miR-23a-3p | novel_circ_0003609 | |
| dre-miR-23a-3p | novel_circ_0003189 | |
| dre-miR-23a-3p | novel_circ_0001780 | |
| dre-miR-23a-3p | novel_circ_0001954 | |
| dre-miR-23a-3p | novel_circ_0000651 | |
| dre-miR-23a-3-5p | novel_circ_0002534 | |
| dre-miR-23a-3-5p | novel_circ_0002872 | |
| dre-miR-23a-3-5p | novel_circ_0001272 | |
| dre-miR-23b | novel_circ_0002482 | |
| dre-miR-23b | novel_circ_0002117 | |
| dre-miR-23b | novel_circ_0003609 | |
| dre-miR-23b | novel_circ_0003189 | |
| dre-miR-23b | novel_circ_0001780 | |
| dre-miR-23b | novel_circ_0001954 | |
| dre-miR-23b | novel_circ_0000651 | |
| dre-miR-23b-5p | novel_circ_0002514 | |
| dre-miR-23b-5p | novel_circ_0002653 | |
| dre-miR-23b-5p | novel_circ_0003599 | |
| dre-miR-23b-5p | novel_circ_0003168 | |
| dre-miR-23b-5p | novel_circ_0002973 | |
| dre-miR-23b-5p | novel_circ_0000577 | |
| dre-miR-24 | novel_circ_0002911 | |
| dre-miR-24 | novel_circ_0002766 | |
| dre-miR-24 | novel_circ_0000778 | |
| dre-miR-24 | novel_circ_0000192 | |
| dre-miR-24 | novel_circ_0001258 | |
| dre-miR-24 | novel_circ_0001022 | |
| dre-miR-24b-3p | novel_circ_0002911 | |
| dre-miR-24b-3p | novel_circ_0002766 | |
| dre-miR-24b-3p | novel_circ_0000778 | |
| dre-miR-24b-3p | novel_circ_0000192 | |
| dre-miR-24b-3p | novel_circ_0001258 | |
| dre-miR-24b-3p | novel_circ_0001022 | |
| dre-miR-29a | novel_circ_0002153 | |
| dre-miR-29a | novel_circ_0003879 | |
| dre-miR-29a | novel_circ_0001059 | |
| dre-miR-29b | novel_circ_0002153 | |
| dre-miR-29b | novel_circ_0003879 | |
| dre-miR-29b | novel_circ_0001059 | |
| dre-miR-30e-5p | novel_circ_0003609 | |
| dre-miR-338-5p | novel_circ_0002674 | |
| dre-miR-365 | novel_circ_0004146 | |
| dre-miR-455-5p | novel_circ_0001659 | |
| dre-miR-455-3p | novel_circ_0003865 | |
| dre-miR-455-3p | novel_circ_0001954 | |
| dre-miR-455-3p | novel_circ_0001979 | |
| dre-miR-455-3p | novel_circ_0004136 | |
| dre-miR-455-3p | novel_circ_0000724 | |
| dre-miR-455-3p | novel_circ_0000482 | |
| dre-miR-455-3p | novel_circ_0001528 | |
| dre-miR-456 | novel_circ_0003842 | |
| dre-miR-456 | novel_circ_0003853 | |
| dre-miR-456 | novel_circ_0002872 | |
| dre-miR-456 | novel_circ_0001373 | |
| dre-miR-456 | novel_circ_0001094 | |
| dre-miR-458-3p | novel_circ_0003583 | |
| dre-miR-489 | novel_circ_0000473 | |
| dre-miR-722 | novel_circ_0003372 | |
| dre-miR-722 | novel_circ_0003246 | |
| dre-miR-722 | novel_circ_0002842 | |
| dre-miR-7a | novel_circ_0003344 | |
| dre-miR-7a | novel_circ_0002999 | |
| dre-miR-7a | novel_circ_0001744 | |
| dre-miR-7a | novel_circ_0001937 | |
| dre-miR-7a | novel_circ_0000764 | |
| dre-miR-9-5p | novel_circ_0002419 | |
| dre-miR-9-5p | novel_circ_0002516 | |
| dre-miR-9-5p | novel_circ_0002534 | |
| dre-miR-9-5p | novel_circ_0002117 | |
| dre-miR-9-5p | novel_circ_0003842 | |
| dre-miR-9-5p | novel_circ_0003247 | |
| dre-miR-9-5p | novel_circ_0002999 | |
| dre-miR-9-5p | novel_circ_0002869 | |
| dre-miR-9-5p | novel_circ_0002031 | |
| dre-miR-9-5p | novel_circ_0000636 | |
| dre-miR-9-5p | novel_circ_0000182 | |
| dre-miR-9-5p | novel_circ_0001544 | |
| dre-miR-9-3p | novel_circ_0002305 | |
| novel_10 | novel_circ_0002482 | |
| novel_10 | novel_circ_0002338 | |
| novel_10 | novel_circ_0003319 | |
| novel_10 | novel_circ_0001784 | |
| novel_10 | novel_circ_0001937 | |
| novel_10 | novel_circ_0001258 | |
| novel_10 | novel_circ_0001267 | |
| novel_10 | novel_circ_0001054 | |
| novel_10 | novel_circ_0001071 | |
| novel_10 | novel_circ_0001544 | |
| novel_101 | novel_circ_0002506 | |
| novel_101 | novel_circ_0003775 | |
| novel_101 | novel_circ_0002744 | |
| novel_101 | novel_circ_0002865 | |
| novel_101 | novel_circ_0002869 | |
| novel_101 | novel_circ_0000741 | |
| novel_101 | novel_circ_0001200 | |
| novel_101 | novel_circ_0001019 | |
| novel_101 | novel_circ_0001041 | |
| novel_101 | novel_circ_0001659 | |
| novel_101 | novel_circ_0001544 | |
| novel_102 | novel_circ_0000456 | |
| novel_1021 | novel_circ_0002395 | |
| novel_1021 | novel_circ_0003599 | |
| novel_1021 | novel_circ_0003098 | |
| novel_1021 | novel_circ_0002744 | |
| novel_1021 | novel_circ_0002872 | |
| novel_1021 | novel_circ_0004136 | |
| novel_1021 | novel_circ_0000182 | |
| novel_1021 | novel_circ_0001370 | |
| novel_1021 | novel_circ_0001391 | |
| novel_1021 | novel_circ_0001439 | |
| novel_1021 | novel_circ_0001528 | |
| novel_1021 | novel_circ_0001569 | |
| novel_1083 | novel_circ_0003828 | |
| novel_1083 | novel_circ_0001637 | |
| novel_113 | novel_circ_0004265 | |
| novel_113 | novel_circ_0003966 | |
| novel_113 | novel_circ_0002744 | |
| novel_113 | novel_circ_0000789 | |
| novel_127 | novel_circ_0002740 | |
| novel_127 | novel_circ_0002203 | |
| novel_127 | novel_circ_0001907 | |
| novel_127 | novel_circ_0000636 | |
| novel_127 | novel_circ_0000577 | |
| novel_128 | novel_circ_0003210 | |
| novel_145 | novel_circ_0004265 | |
| novel_145 | novel_circ_0003966 | |
| novel_145 | novel_circ_0002744 | |
| novel_145 | novel_circ_0000789 | |
| novel_157 | novel_circ_0002732 | |
| novel_157 | novel_circ_0002737 | |
| novel_157 | novel_circ_0000788 | |
| novel_158 | novel_circ_0002338 | |
| novel_158 | novel_circ_0001258 | |
| novel_158 | novel_circ_0001298 | |
| novel_163 | novel_circ_0002117 | |
| novel_163 | novel_circ_0003210 | |
| novel_163 | novel_circ_0000731 | |
| novel_163 | novel_circ_0000250 | |
| novel_200 | novel_circ_0002732 | |
| novel_226 | novel_circ_0002350 | |
| novel_226 | novel_circ_0003142 | |
| novel_226 | novel_circ_0001979 | |
| novel_227 | novel_circ_0002305 | |
| novel_227 | novel_circ_0003459 | |
| novel_227 | novel_circ_0002865 | |
| novel_23 | novel_circ_0002922 | |
| novel_23 | novel_circ_0001439 | |
| novel_231 | novel_circ_0004265 | |
| novel_231 | novel_circ_0002119 | |
| novel_231 | novel_circ_0002398 | |
| novel_231 | novel_circ_0003853 | |
| novel_231 | novel_circ_0003744 | |
| novel_231 | novel_circ_0003168 | |
| novel_231 | novel_circ_0003247 | |
| novel_231 | novel_circ_0001842 | |
| novel_231 | novel_circ_0001907 | |
| novel_231 | novel_circ_0001937 | |
| novel_231 | novel_circ_0001283 | |
| novel_234 | novel_circ_0003891 | |
| novel_234 | novel_circ_0003210 | |
| novel_234 | novel_circ_0002973 | |
| novel_234 | novel_circ_0001819 | |
| novel_255 | novel_circ_0004265 | |
| novel_255 | novel_circ_0003210 | |
| novel_255 | novel_circ_0003246 | |
| novel_255 | novel_circ_0002842 | |
| novel_255 | novel_circ_0002844 | |
| novel_255 | novel_circ_0000473 | |
| novel_255 | novel_circ_0000474 | |
| novel_255 | novel_circ_0000577 | |
| novel_262 | novel_circ_0002695 | |
| novel_262 | novel_circ_0002696 | |
| novel_262 | novel_circ_0002482 | |
| novel_262 | novel_circ_0002260 | |
| novel_262 | novel_circ_0003828 | |
| novel_262 | novel_circ_0003599 | |
| novel_262 | novel_circ_0000456 | |
| novel_262 | novel_circ_0001391 | |
| novel_262 | novel_circ_0001267 | |
| novel_262 | novel_circ_0001272 | |
| novel_262 | novel_circ_0001054 | |
| novel_277 | novel_circ_0003966 | |
| novel_277 | novel_circ_0003221 | |
| novel_277 | novel_circ_0003246 | |
| novel_277 | novel_circ_0001866 | |
| novel_327 | novel_circ_0002203 | |
| novel_327 | novel_circ_0003142 | |
| novel_38 | novel_circ_0003372 | |
| novel_38 | novel_circ_0003245 | |
| novel_38 | novel_circ_0003247 | |
| novel_49 | novel_circ_0003247 | |
| novel_49 | novel_circ_0000789 | |
| novel_49 | novel_circ_0000246 | |
| novel_49 | novel_circ_0000250 | |
| novel_494 | novel_circ_0003221 | |
| novel_494 | novel_circ_0003142 | |
| novel_494 | novel_circ_0003146 | |
| novel_511 | novel_circ_0004267 | |
| novel_530 | novel_circ_0004195 | |
| novel_60 | novel_circ_0003344 | |
| novel_60 | novel_circ_0002999 | |
| novel_60 | novel_circ_0001744 | |
| novel_60 | novel_circ_0001937 | |
| novel_60 | novel_circ_0000764 | |
| novel_605 | novel_circ_0002744 | |
| novel_605 | novel_circ_0002869 | |
| novel_605 | novel_circ_0001784 | |
| novel_605 | novel_circ_0000577 | |
| novel_605 | novel_circ_0001022 | |
| novel_644 | novel_circ_0002482 | |
| novel_644 | novel_circ_0002117 | |
| novel_644 | novel_circ_0003609 | |
| novel_644 | novel_circ_0003189 | |
| novel_644 | novel_circ_0001780 | |
| novel_644 | novel_circ_0001954 | |
| novel_644 | novel_circ_0000651 | |
| novel_645 | novel_circ_0002674 | |
| novel_645 | novel_circ_0003189 | |
| novel_645 | novel_circ_0003245 | |
| novel_645 | novel_circ_0003142 | |
| novel_645 | novel_circ_0000259 | |
| novel_663 | novel_circ_0002395 | |
| novel_663 | novel_circ_0003853 | |
| novel_663 | novel_circ_0003744 | |
| novel_663 | novel_circ_0003142 | |
| novel_663 | novel_circ_0001019 | |
| novel_670 | novel_circ_0002732 | |
| novel_670 | novel_circ_0002453 | |
| novel_670 | novel_circ_0003560 | |
| novel_670 | novel_circ_0003210 | |
| novel_670 | novel_circ_0003246 | |
| novel_670 | novel_circ_0002999 | |
| novel_670 | novel_circ_0000731 | |
| novel_670 | novel_circ_0000764 | |
| novel_670 | novel_circ_0000473 | |
| novel_670 | novel_circ_0000474 | |
| novel_670 | novel_circ_0000180 | |
| novel_674 | novel_circ_0002153 | |
| novel_681 | novel_circ_0002305 | |
| novel_681 | novel_circ_0003560 | |
| novel_681 | novel_circ_0000473 | |
| novel_681 | novel_circ_0000474 | |
| novel_69 | novel_circ_0004267 | |
| novel_781 | novel_circ_0002180 | |
| novel_781 | novel_circ_0003966 | |
| novel_781 | novel_circ_0004136 | |
| novel_781 | novel_circ_0000741 | |
| novel_781 | novel_circ_0000348 | |
| novel_853 | novel_circ_0002490 | |
| novel_87 | novel_circ_0002537 | |
| novel_87 | novel_circ_0002203 | |
| novel_87 | novel_circ_0002338 | |
| novel_87 | novel_circ_0001272 | |
| novel_902 | novel_circ_0000482 | |
| novel_911 | novel_circ_0002305 | |
| novel_911 | novel_circ_0003229 | |
| novel_911 | novel_circ_0002774 | |
| novel_911 | novel_circ_0002774 | |
| novel_911 | novel_circ_0002031 | |
| novel_920 | novel_circ_0002395 | |
| novel_920 | novel_circ_0001784 | |
| novel_920 | novel_circ_0000651 | |
| novel_920 | novel_circ_0001637 | |
| novel_938 | novel_circ_0002537 | |
| novel_938 | novel_circ_0003372 | |
| novel_938 | novel_circ_0001958 | |
| novel_938 | novel_circ_0001959 | |
| novel_938 | novel_circ_0002081 | |
| novel_938 | novel_circ_0001391 | |
| novel_974 | novel_circ_0002117 | |
| novel_987 | novel_circ_0002674 | |
| novel_987 | novel_circ_0002180 | |
| novel_987 | novel_circ_0002338 | |
| novel_987 | novel_circ_0003865 | |
| novel_987 | novel_circ_0003210 | |
| novel_987 | novel_circ_0003221 | |
| novel_987 | novel_circ_0003254 | |
| novel_987 | novel_circ_0003319 | |
| novel_987 | novel_circ_0002922 | |
| novel_987 | novel_circ_0003119 | |
| novel_987 | novel_circ_0002869 | |
| novel_987 | novel_circ_0004002 | |
| novel_987 | novel_circ_0000256 | |
| novel_987 | novel_circ_0001061 | |
